# Pulsed electrolysis for CO_2_ reduction: Techno-economic perspectives

**DOI:** 10.1016/j.isci.2024.110383

**Published:** 2024-06-27

**Authors:** You Lim Chung, Sojin Kim, Youngwon Lee, Devina Thasia Wijaya, Chan Woo Lee, Kyoungsuk Jin, Jonggeol Na

**Affiliations:** 1 Department of Chemical Engineering and Materials Science, Ewha Womans University, Seoul 03760, Republic of Korea; 2 Graduate Program in System Health Science and Engineering Ewha Womans University, Seoul 03760, Republic of Korea; 3 Department of Chemistry, Korea University, Seoul 02841, Republic of Korea; 4 Department of Chemistry, Kookmin University, Seoul 02707, Republic of Korea

**Keywords:** Chemical engineering, Catalysis, Electrochemistry

## Abstract

Pulsed electrolysis has emerged as a promising approach to CO_2_ reduction, offering a simple method to adjust product selectivity and enhance operational stability. However, conceptually applying the dynamic pulse operation process on a large scale highlights its differences when compared to conventional electrolysis processes, impacting the economic feasibility of the process. We discuss the influence of pulsed electrolysis on surface reaction mechanisms and the simulation of changes at both the continuum and smaller scales through computational modeling. Additionally, we point out considerations for applying pulsed CO_2_ electrolysis to a large-scale process and assess their economic implications, comparing pulsed electrolysis with constant electrolysis.

## Graphical abstract

**Figure:**
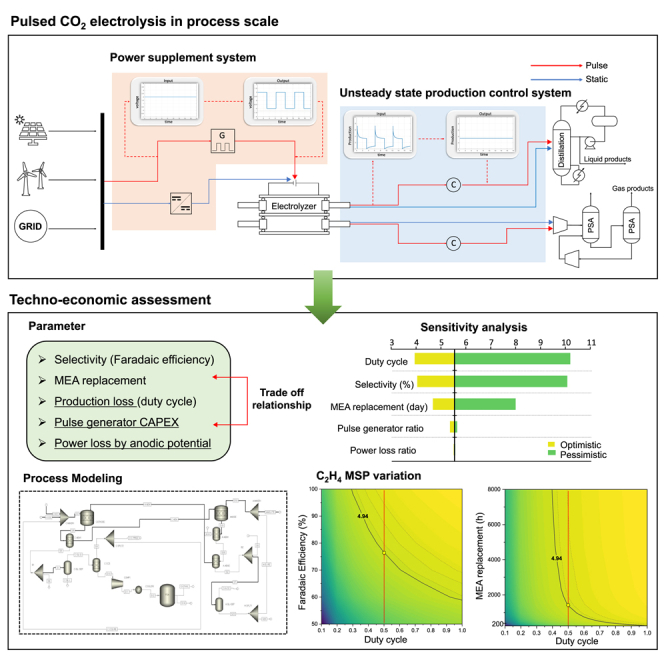

## Introduction

The rapid increase in CO_2_ emissions accompanying recent industrialization has prompted an urgent response to greenhouse gases. Consequently, the effective conversion and utilization of CO_2_ have emerged as crucial challenges for sustainable energy and environmental protection. The electroreduction of CO_2_ has been pursued as a promising solution to close the anthropogenic carbon cycle by mitigating carbon emission while producing chemical fuels for energy storage. In the past decade, significant advancements in catalyst design, cell design, and electrochemical processes have been reported.^1^^,^^2^^,^^3^ However, due to the high requirements of cost-competitive CO_2_ electroreduction (>2000 h of continuous operation with >200 mA cm^−2^ current density and high selectivity^4^^,^^5^^,^^6^), highly active, selective, and stable catalysts and electrocatalysis processes are needed for implementation on an industrial scale.

Over the past few decades, the prevalent use of the three-electrode H-cell configuration in CO_2_ electroreduction has markedly progressed catalyst refinement and brought to light crucial knowledge of reaction mechanisms. Nevertheless, the low CO_2_ concentration and its slow diffusion impede the reaction kinetics, posing obstacles to the implementation of large-scale CO_2_ conversion.^7^ By using continuous flow cells with gas diffusion electrodes (GDE), a high-rate CO_2_ electroreduction can be achieved, as CO_2_ can be directly supplied to the catalyst surface.^8^^,^^9^ However, common stability issues—such as catalyst poisoning,^10^ surface reconstruction,^11^ and salt precipitation^12^—often hinder the longevity of the CO_2_ reduction process. In addition, applying a high current quickly deteriorates the electrode, causing the selectivity to shift to unfavorable products, such as H_2_. Therefore, much effort has been made in catalyst engineering or process design to overcome this issue.^13^^,^^14^

Recently, pulsed electrolysis emerged as a promising technique, enabling stable high-rate CO_2_ electroreduction due to its dynamic process induced during electrolysis. This approach, characterized by its simplicity, does not need intricate syntheses or pretreatments and can induce changes in the reaction environment. With the proper design of the applied potential and cycling period, stable performance can be achieved for up to 200 h with >100 mA cm^-^^2^ current density^15^^,^^16^ with only minor degradation in the observed product selectivity. In addition, pulsing the potential allows for the proper regulation of the adsorbate coverage on the catalyst surface and thus enables the fine-tuning of product selectivity, especially for Cu catalysts, which are known for their ability to produce multi-carbon products from CO_2_ electroreduction.^17^ However, due to a limited understanding of the pulsed electrolysis mechanism, adjusting the pulse profile appropriately can be challenging. This limitation can be overcome by combining advancements in *in situ* and *operando* spectroscopy with computational modeling.^18^^,^^19^

Pulsed CO_2_ electrolysis enhances selectivity and stability over conventional CO_2_ electroreduction, but the absence of product generation while the anodic potential is applied leads to production loss. Additionally, there are differences between the pulsed and conventional electrochemical CO_2_ reduction reactions (CO_2_RRs), such as the method of supplying power to the electrolyzer and the fact that the products generated in the electrolyzer exhibit non-steady-state characteristics. Hence, it is crucial to evaluate pulsed CO_2_ electrolysis economic viability when applying it at an industrial scale. However, there is no existing research related to the techno-economic assessment of the pulsed electrolysis system. In this work, we summarize the current status, recent progress, and challenges of utilizing pulsed electrolysis for CO_2_ reduction. First, we explain the concept of pulsing electrolysis for CO_2_ reduction, followed by its important effects on the CO_2_ electrolysis process. Second, we summarize recent advancements in the computational methods aiding in the pulse profile design and understanding of the mechanism behind pulsed CO_2_ electrolysis. Third, we outline the procedural differences between pulsed electrolysis applied at an industrial scale and perform a techno-economic analysis.

## Pulse-assisted electrochemical CO_2_ reduction

Extensive efforts have been dedicated to identifying optimal catalysts for achieving selectivity and catalytic efficiency in CO_2_ reduction. For instance, Hori et al.^20^ investigated various metal electrodes and found that only copper electrodes are able to produce multi-carbon products such as hydrocarbons and alcohols. While copper and copper-based electrocatalysts have demonstrated exceptional performance in CO_2_ reduction, they face several critical challenges, such as catalyst degradation during long-term electrolysis due to poisoning and aggregation issues.^21^^,^^22^^,^^23^^,^^24^^,^^25^

To address these challenges, pulse-assisted CO_2_ reduction has gained widespread attention. Pulse-assisted electrocatalysis can enhance selectivity through surface engineering and improve long-term stability. In this section, we begin by introducing the fundamental concepts and recent advances in pulse-assisted CO_2_ reduction reactions. Subsequently, we explore several hypotheses that have been put forward to elucidate the effects of pulse systems in CO_2_ reduction catalysis, including (1) catalyst poisoning inhibition, (2) surface reconstruction, (3) surface coverage rearrangement, and (4) mass transport limitation mitigation.

## Pulse-assisted electrocatalysis

**Figure 1:**
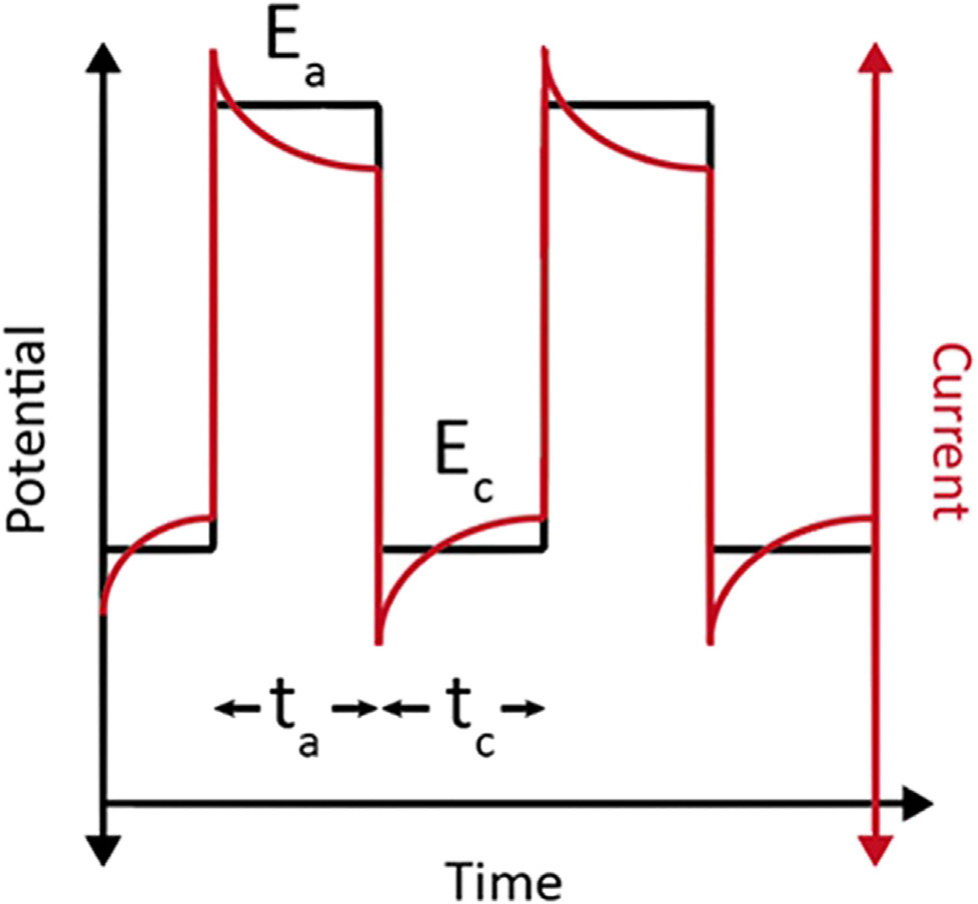
Example pulse profile showing the anodic potential (E_a_) and time period (t_a_) and the cathodic potential (E_c_) and time period (t_c_) in black (left axis) along with the current in red (right axis) over time

“Pulsed electrolysis” refers to dynamic electrochemical processes in which the applied potentials or current periodically changes in a designated sequence (Figure 1). While conventional electrolysis employs a constant current (chronopotentiometry) or constant potential (chronoamperometry), diverse pulse waveforms, such as triangular, sawtooth, sinusoidal, or asymmetric waves, can be utilized for pulsed electrolysis. Compared to other pulse profiles such as triangle-shaped or sinusoidal-shaped ones, the square profile can easily investigate the effects of various electrochemical parameters, such as applied anodic/cathodic potentials, and duration of applied potentials. Facile control of the square pulse profile can also provide good reproducibility. For instance, in Engelbrecht’s work, the authors confirmed the relation between the applied cathodic potential and CO_2_RR selectivity square pulse profile. The cathodic potential under −1.7 V exhibited a significant suppression of HER.^26^ In addition, the authors confirmed the different products were observed depending on the anodic potential. Ethylene was maximally formed at −0.33 V and methane at −0.18 V, respectively. Likewise, In Bui’s work, the authors verified the occurrence of dynamic changes in pH and CO_2_ concentration during square pulse electrolysis using their developed time-dependent continuum model and confirmed that elevated pH influences an increase in the selectivity of C_2+_ products.^27^ While square pulse offers advantages in product selectivity and faradaic efficiency (FE), it has the drawback of potentially having higher ripple values compared to other pulse profiles, which could lead to power efficiency losses.^28^^,^^29^ Dobó et al. confirmed that square waveform exhibits the highest ripple factor compared to other pulse profiles (triangle, sawtooth, and sine), which was identified as one of the parameters affecting efficiency losses in water electrolysis.^28^ The sawtooth waveform, which linearly increases and then decreases voltage, can be utilized for studying electron transfer chemical reaction.^30^ The sinusoidal waveform, oscillating between two extremes over time, is commonly utilized in impedance research.^31^^,^^32^ Among these, the most extensively used waveform for pulsed electrolysis is the square wave due to their advantages in terms of product selectivity and FE. The key parameters in pulse electrolysis encompass the applied potentials, anodic (E_a_) and cathodic (E_c_), along with the pulse times, respectively, denoted as the anodic (t_a_) and cathodic (t_c_) pulse times. As displayed in Figure 1, the pulse height indicates the size of pulse step. Generally, the “anodic” potential denotes the most positive potential within the pulse sequence, with exceptions where it represents the potential undergoing anodic current. Throughout the anodic pulse sequence, the anodic potential governs whether the catalyst undergoes oxidation or solely experiences double-layer rearrangement.

Further detailing the double-layer rearrangement, the current profile undergoes three distinct phases when the cathodic potential is applied. Initially, a non-faradaic charging process occurs, causing the charging current to exponentially decrease over time, as described by Equation 1^33^:

(Equation 1) $$E=iR_{s}+\frac{q}{C_{d}},i=\frac{E}{R_{s}}e^{-t/R_{s}C_{d}}$$

where E, $i,R_{s}$, $C_{d}$, and q are potential, current, solution resistance, capacitance of the double layer, and charge. The current equation depicted in Equation 1 can be derived as a general expression for the charge q as a function of the voltage E_c_ applied to the capacitor. The total voltage across the resistor (E_R_) and capacitor (E_c_) always equals the applied voltage. By substituting *i* = dq/dt, rearranging, and assuming the initial discharge of the capacitor, differentiating leads to the obtained equation. Subsequently, the faradaic current resulting from redox reaction in a diffusive system undergoes a transition from non-faradaic to conforming to the t^−1/2^ behavior, following the Cottrell equation (Equation 2)^33^:

(Equation 2) $$i=\frac{n\text{FA}c_{j}^{0}\sqrt{D_{j}}}{\sqrt{\pi t}}$$

where n is the number of electrons involved in the reaction, **F** is the Faraday constant, *A* is the area of the electrode, $c_{j}^{0}$ is the initial concentration of species *j*, and *D*_*j*_ is the diffusion coefficient of species *j*. Since electrochemical reactions take place at the electrode surface, the movement of reactants toward the electrode surface is crucial. Consequently, current is determined by diffusion, and this behavior is represented by the Cottrell equation. When a potential is applied for the extended period, current decreases due to catalyst degradation, reshaping, poisoning, etc.^22^^,^^23^^,^^24^^,^^25^^,^^34^^,^^35^^,^^36^^,^^37^^,^^38^^,^^39^ To sum up, when a potential is applied, the current follows Equation 1 during the period of filling the electrical double layer. Subsequently, as this process concludes, the current transitions to follow the Cottrell equation (Equation 2) until reaching the limiting value. For this reason, cathodic and anodic potential pulse time (t_c_ and t_a_) is important in pulsed electrolysis. Adjusting the key parameters (E_c_, E_a_, t_c_, and t_a_) can modulate the reaction environment and thus influence the product selectivity during the electrolysis.

## Effects of pulse electrocatalysis on CO_2_ reduction

### Inhibition of catalyst poisoning

During CO_2_RRs, catalyst surfaces undergo continuous changes. Occasionally, during the reaction, the catalyst surfaces on electrodes become poisoned, leading to severe deactivation.^35^^,^^40^ There are various reasons for the occurrence of poisoning events such as C or CO poisoning, trace metal ion impurities, contamination, salt formation, etc.^34^^,^^35^^,^^36^^,^^37^^,^^38^^,^^39^ For example, Akhade et al. demonstrated that the utilization of Pt, Ni, Co, and Fe electrodes in the CO_2_RR does not lead to the production of hydrocarbons due to CO poisoning.^36^ The authors confirmed through the thermodynamic analysis that the presence of a high coverage of CO∗ blocks the H∗ sites, thereby hindering the formation of C-H bonding sites. Also, surface active reagents contained in the solvent, impurities such as dissolved glassware, and trace metal ion can poison the CO_2_ reduction catalysts.^34^^,^^35^^,^^37^^,^^38^^,^^39^ In the poisoning species deposition occurs on the electrode; the activity of catalysts, partial current density values, and target product production can drop significantly within minutes of beginning electrolysis due to the deposition of poisoning species on the electrode. To maintain high catalyst activity, it is essential to suppress these undesired poisoning events. Electrochemical pulse methods have been known to effectively prevent the deposition of poisoning species, thus enhancing the stability of catalysts.

**Figure 2:**
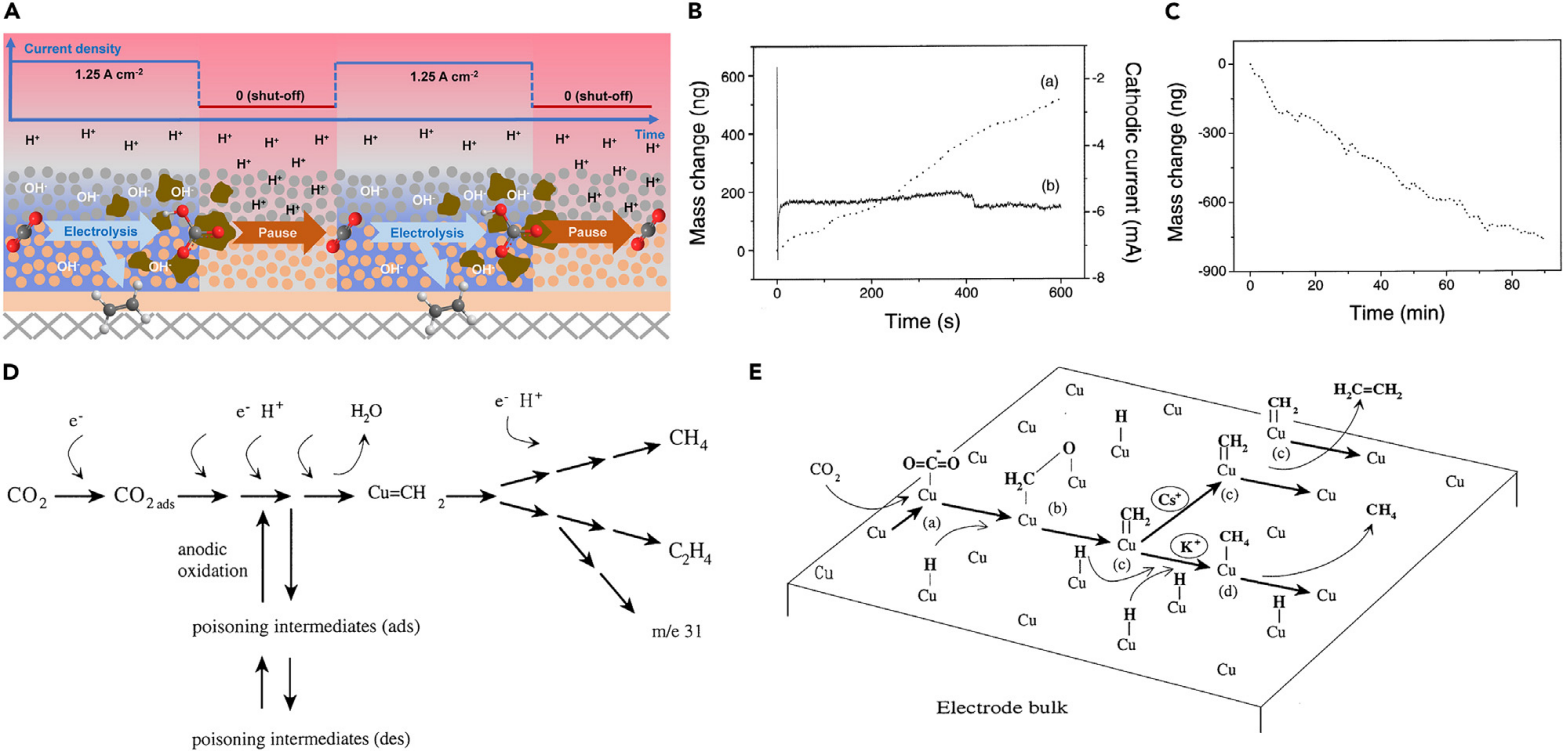
Strategies for inhibiting the surface poisoning of the electrode (A) A schematics of the pause system (reprinted with permission from Xu et al.^41^ Copyright 2022, Elsevier). The mass change of Cu using a constant (B) and pulsed (C) potential (reprinted with permission from Lee et al.^22^ Copyright 2001, Elsevier). Reaction pathway (D) and overview scheme (E) of CO_2_RRs at the copper electrode surface (reprinted with permission from Park et al.^23^ Copyright 1997, Elsevier).

In alkaline conditions, reaction of CO_2_ with hydroxide results in the formation of both carbonate and bicarbonate salts precipitating onto the cathode, leading to unintended carbon loss and, potentially, device failure. This problem was resolved by operating a modified CO_2_RR electrolysis in acidic media. Xu et al.^41^ introduced a “pause” electrolysis system, characterized by intermittent shut-off periods during continuous electrolysis, as depicted in Figure 2A. A three-dimensional gas diffusion electrode was employed under acidic conditions to increase the number of active sites and achieve high CO_2_RR current density and stable CO_2_RR activity. The authors claimed that the 20 min paused period served to mitigate salt formation by allowing reactions with adjacent H^+^ ions. The “pause” provided the time needed for protons to diffuse back to the interface and for bicarbonate to leave the surface, whereas constant electrolysis only consumes H^+^, resulting in an alkaline interface. The recovery of H^+^ concentration regenerates local CO_2_ concentration, increasing C_2_H_4_ FE. Furthermore, Xu et al.^42^ proposed the concept of self-cleaning CO_2_ reduction systems designed to avert the formation of solid potassium carbonate salts. These utilized regeneration periods with potentials that reduce the reaction rate to nearly zero, which prevents hydroxide formation and moves carbonate ions away from the cathode. The authors used a COMSOL Multiphysics simulation to predict the ion concentration profiles that exceed the solubility limit and confirmed that on a timescale of minutes, salt precipitation is unavoidable in steady state systems. The authors also simulated carbonate concentrations under different regeneration times and different migration conditions and confirmed that a regeneration step can keep carbonate concentrations below the solubility limit and prevent salt formation. In this system, the authors claimed that a regeneration interval prevented salt production, allowing for long-term stability of operation.

To explore the effects of the pulse technique on the formation of poisoning species, Lee et al.^22^ investigated amorphous carbon formation using *in situ* electrochemical quartz crystal microbalance (EQCM). When subjected to chronoamperometry at −2.1 V for 10 min (potentiostatic condition), a change in the Cu cathode mass was observed (Figure 2B). This change was attributed to the absorption of amorphous carbon on the electrode surface, resulting in a significant decrease of catalytic activity over time. In contrast, “potential modulation electrolysis” (pulsed electrolysis) provoked a structural change in the electrode by the dynamic electro-dissolution of Cu (Figure 2C). The authors claimed that the formation of Cu_2_O by applying the potential modulation electrolysis effectively prevented the poisoning of carbon on the Cu electrode. Friebe et al.^23^ identified the catalyst deactivation pathway using differential electrochemical mass spectrometry (DEMS), as shown in Figures 2D and 2E. The authors also considered the formation of the undesirable absorbate as the main pathway of catalyst deactivation. To address the issue, the authors adopted pulse electrolysis. Applying an anodic pulse at 0.05 V vs. standard hydrogen electrode (SHE) for 10 s resulted in the oxidation of adsorbate species, which was found to suppress the deactivation.

### Surface reconstruction

As the CO_2_RR reactions occur on the electrode surface, various factors, including catalyst deposition, the Cu oxidation state, and the surrounding electrode environment, can greatly influence the kinetics of the reaction. As with surface poisoning inhibition, surface engineering by pulsed electrolysis has been studied.

**Figure 3:**
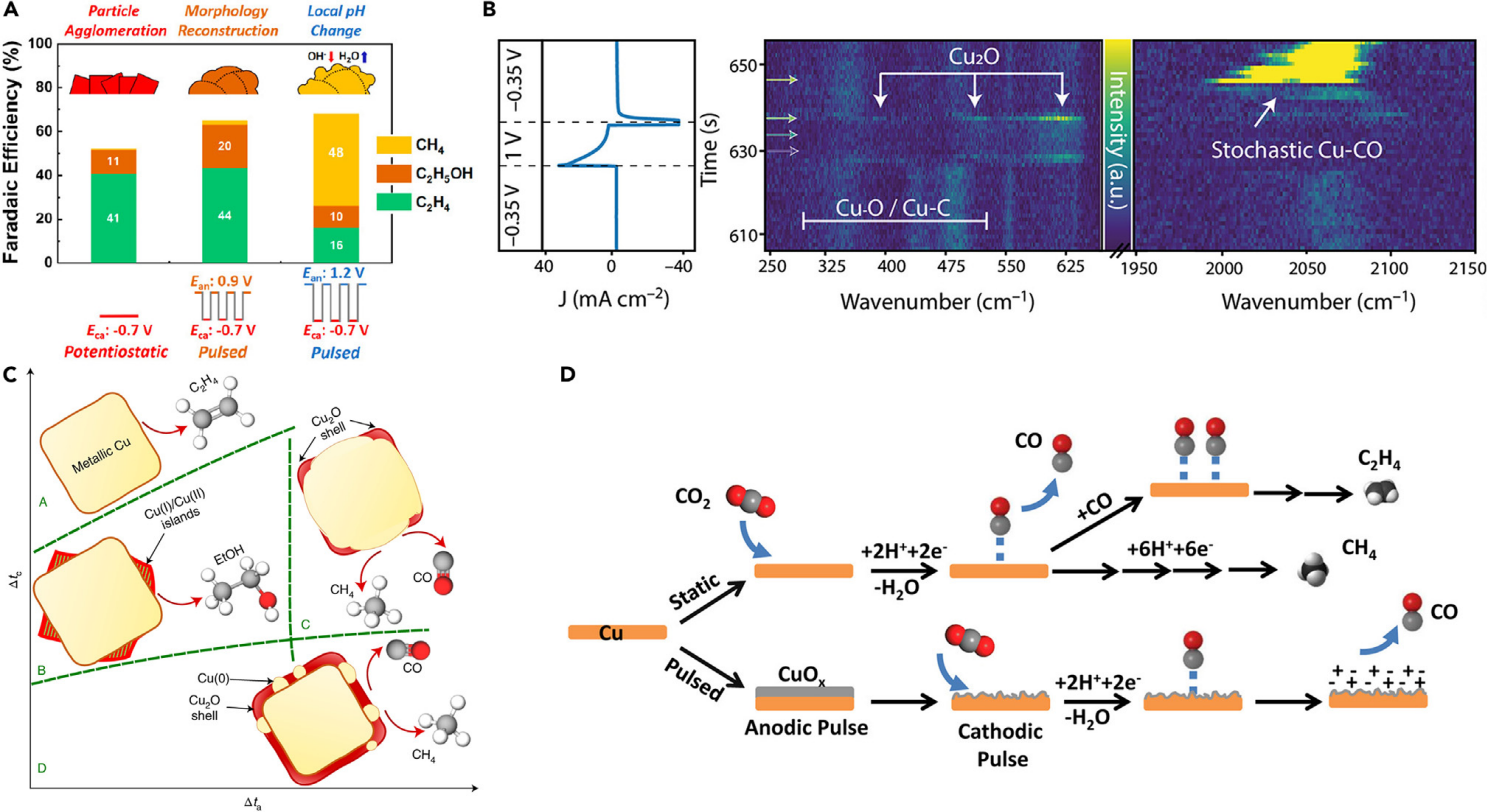
Surface reconstruction during pulse-assisted CO_2_ reduction catalysis (A) Structural modifications and products resulting from different electrochemical methods (reprinted with permission from Jeon et al.^43^ Copyright 2021, American Chemical Society). (B) TR-SERS data during pulsed electrolysis, showing stochastic CO formation after finishing the anodic pulse (reprinted with permission from Ruiter et al.^44^ Copyright 2022, American Chemical Society). (C) Modifications to the catalyst structure and composition during the cathodic pulse (reprinted with permission from Timoshenko et al.^46^ Copyright 2022, Springer Nature). (D) A depiction of catalyst surfaces during static and pulsed electrolysis (reprinted with permission from Kumar et al.^48^ Copyright 2016, American Chemical Society).

Jeon et al.^43^ monitored the product distribution under different pulse profiles (Figure 3A). Interestingly, the authors revealed that anodic potentials in the pulse sequence can also affect the CO_2_RR selectivity. When the anodic potential is applied under 0.9 V, C_2_H_4_ was found to be the main product, whereas CH_4_ was the main product when the anodic potential was raised to 1.0 V. *Operando* spectroscopy (XAS, SERS) was used for monitoring the shape of the catalysts and chemical state changes, and *ex situ* SEM and TEM analyses were conducted to monitor the phase change during electrolysis. This showed that Cu nanocubes (NCs) morphology undergoes irreversible changes during pulsed electrolysis that contributes to enhancing C_2_ product selectivity. Moreover, at the E = 1.2 V condition, CH_4_ formation is affected by local pH conditions, the reasons for which we describe further in section improvements in catalytic stability. To explore the electrode surface during the reaction, Ruiter et al.^44^ identified the formation of “stochastic CO” right after applying an anodic pulse using time-resolved Raman spectroscopy (TR-SERS). This method can monitor electrode surface reconstruction and the formation of CO intermediates, as displayed in Figure 3B. By coupling cyclic voltammetry (CV) and TR-SERS experiments, they observed four distinct regions that confirmed the presence of redox peaks involved in the reaction. Using TR-SERS during the pulsed electrolysis, which mimicked the CV cycle, confirmed the presence of stochastic CO. They suggested that the oxidation of the electrode’s surface during the anodic pulse can enhance the dominance of CO formation at lower overpotentials. Li et al. revealed the pulsed induced intermediate control using the fast time-resolved *in situ* surface-enhanced Raman spectroscopy. The authors monitored the CO_ads_ peaks using TR-SERS and confirmed that CO_ads_ is an important factor in controlling the selectivity of CO_2_RR. Depending on the population of CO_ads_, the Cu_x_O/Cu surface can regulate the C_2+_/C_1_ ratio of CO_2_RR. Manipulating anodic potential and pulse duration both mitigated surface poisoning and heightened selectivity toward C_2+_ products.^45^ Anodic potential was adjusted within a range of enabling oxidation of the catalyst. Product selectivity also varies depending on the oxidation state of the catalyst. Timoshenko et al.^46^ explored diverse catalyst surface structures and oxidation states, unveiling four distinct domains dependent on pulse times (t_a_, t_c_) (Figure 3C). The authors claimed that the manipulation of potential pulses and metallic species components, along with distinct Cu oxidation states, selectively yielded the C_2_ products. Xu et al.^47^ also suggested that an *in situ* periodic regeneration of catalyst (PR-C) strategy that resulted in a mixture of Cu^+^ and Cu^0^ states could promote C_2_ production. They highlighted that an abundance of Cu^+^ species contributed to facilitating the C–C coupling, pinpointing a 0.41 oxidation state as the optimal configuration for achieving a 70.3% FE. On the other hand, Kumar et al.^48^ applied short pulse times (<50 ms) to exclusively produce syngas products with varying H_2_:CO ratios, deviating from prior studies. They postulated that alterations in the electric field, induced by charging the double layer near the electrode, could influence the binding energy of absorbed CO intermediates (Figure 3D).

### Surface adsorbates rearrangement

In the context of surface dynamics, the modulation of surface adsorbates, including H_ads_ and CO_ads_, is a key factor in directing product selectivity in pulsed electrochemical CO_2_RRs.^49^ Shiratsuchi et al.,^50^ using silver electrodes, and Ishimaru et al.,^51^ using Cu/Ag Alloy electrodes, both reported product selectivity depending on anodic potential. Under anodic potentials less negative than −0.4 V, H_ads_ detachment from the electrode fostered the formation of CO and HCOOH, and the strength of the negative potential dictated the outcome of CO_ads_-induced products, with more negative potentials favoring CH_4_, C_2_H_4_, and C_2_H_5_OH. Kimura et al.^24^ confirmed the product selectivity in CO reduction reaction (CORR). To understand the mechanism behind this selectivity, they showed that pulsed potentials could suppress the hydrogen evolution reaction (HER), thereby enhancing selectivity by detaching the H_ads_ and CO_ads_ attached to the surface.

**Figure 4:**
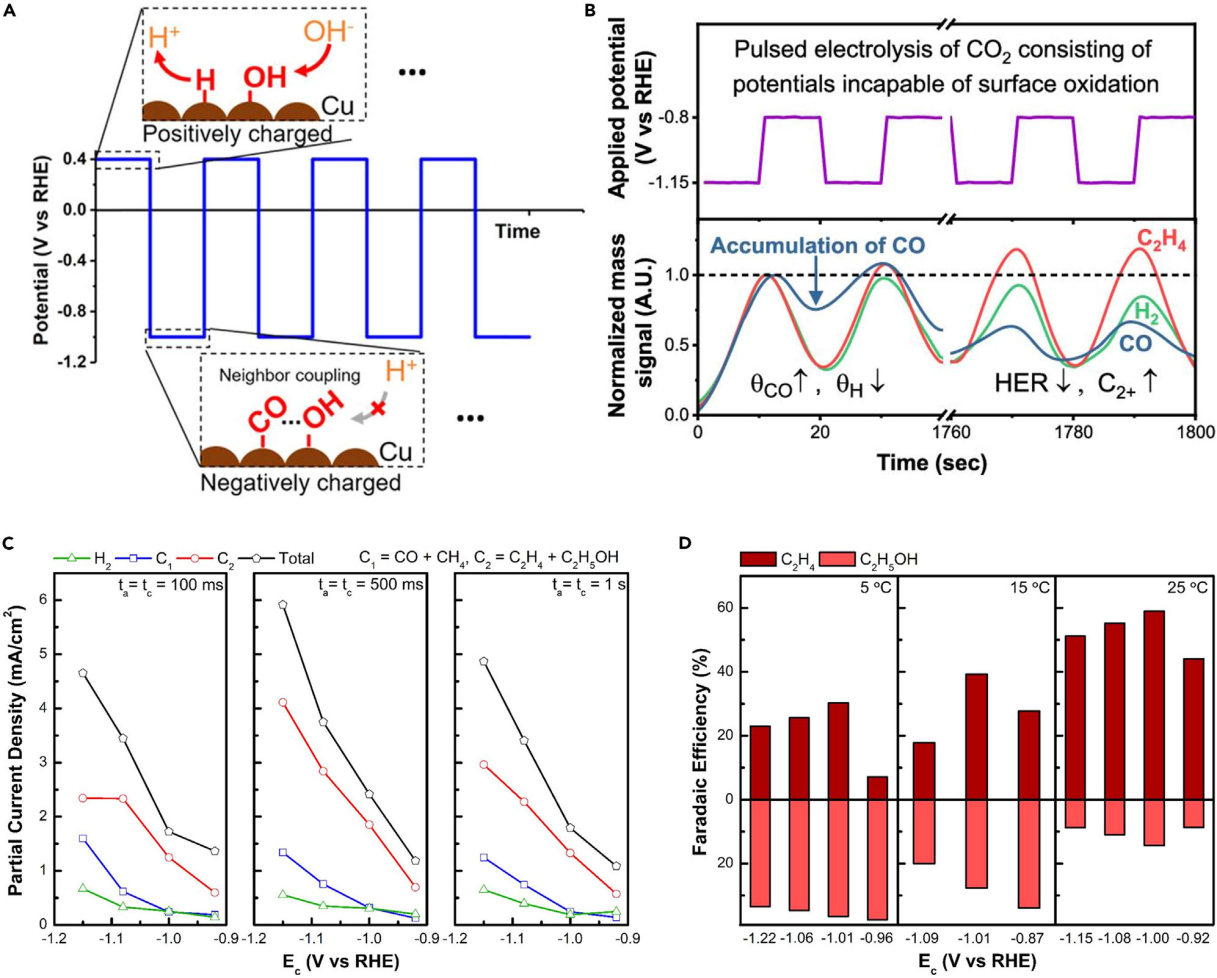
Surface coverage rearrangement during pulse-assisted CO_2_ reduction catalysis (A) Dynamic surface coverage during pulsed electrolysis (reprinted with permission from Kimura et al.^25^ Copyright 2020, American Chemical Society). (B) A depiction of real-time mass signals during pulsed electrolysis (reprinted with permission from Kim et al.^52^ Copyright 2020, American Chemical Society). (C) The partial current densities of CO_2_RR products at different E_c_ values and pulse times (t_a_ = t_c_). (D) The FEs of C_2_ product at different E_c_ values and temperatures (reprinted with permission from Tang et al.^53^ Copyright 2021, American Chemical Society).

From a different adsorbate perspective, applying the anodic potential promoted the formation of OH_ads_, inducing a high pH environment conducive to near-neighbor coupling with CO_ads_ (Figure 4A).^25^ By measuring the product signal using DEMS, Kim et al.^52^ confirmed the product distribution under the pulsed method. DEMS results showed that the pulsed method can facilitate electrolysis with high local CO_2_ concentration accumulated during the −0.8 V vs. RHE period and increase the concentrations of CO_ads_/H_ads_ on the surface, which contributes the increase of C_2+_ product selectivity and decrease in H_2_ selectivity (Figure 4B). Tang et al.^53^ expanded this with partial current density data to adjust the pulse length, showing that the H_2_ and C_1_ product’s partial current densities decreased, while C_2_ partial current densities increased, with controlling pulse lengths compared to their previous constant potential condition (Figure 4C).^54^ They further proposed that temperature acts as a control factor for selectivity between ethylene and ethanol, as depicted in Figure 4D. Apparently related to the reduction rate of Cu species, C_2_H_4_ was preferentially produced at higher temperatures, and C_2_H_5_OH was preferentially produced at lower temperatures. Similarly, Duff et al.^49^ suggested a correlation between oxygenated products and OH_ads_ on various Cu surfaces. To sum up, in the case of CO_2_ reduction catalysis, the population of surface adsorbates can largely affect the product selectivity. In this regard, the researchers reported that the pulses method selectively enhances the selectivity and efficiency of CO_2_RR products by promoting desorption or adsorption depending on the type of surface adsorbates. It has been proposed that H_ads_ facilitates the formation of CO_ads_, collectively enhancing C_2_ product selectivity, whereas OH_ads_ contributes to creating a higher pH environment near the electrode, effectively suppressing the HER.

### Mass transport limitation mitigation

**Figure 5:**
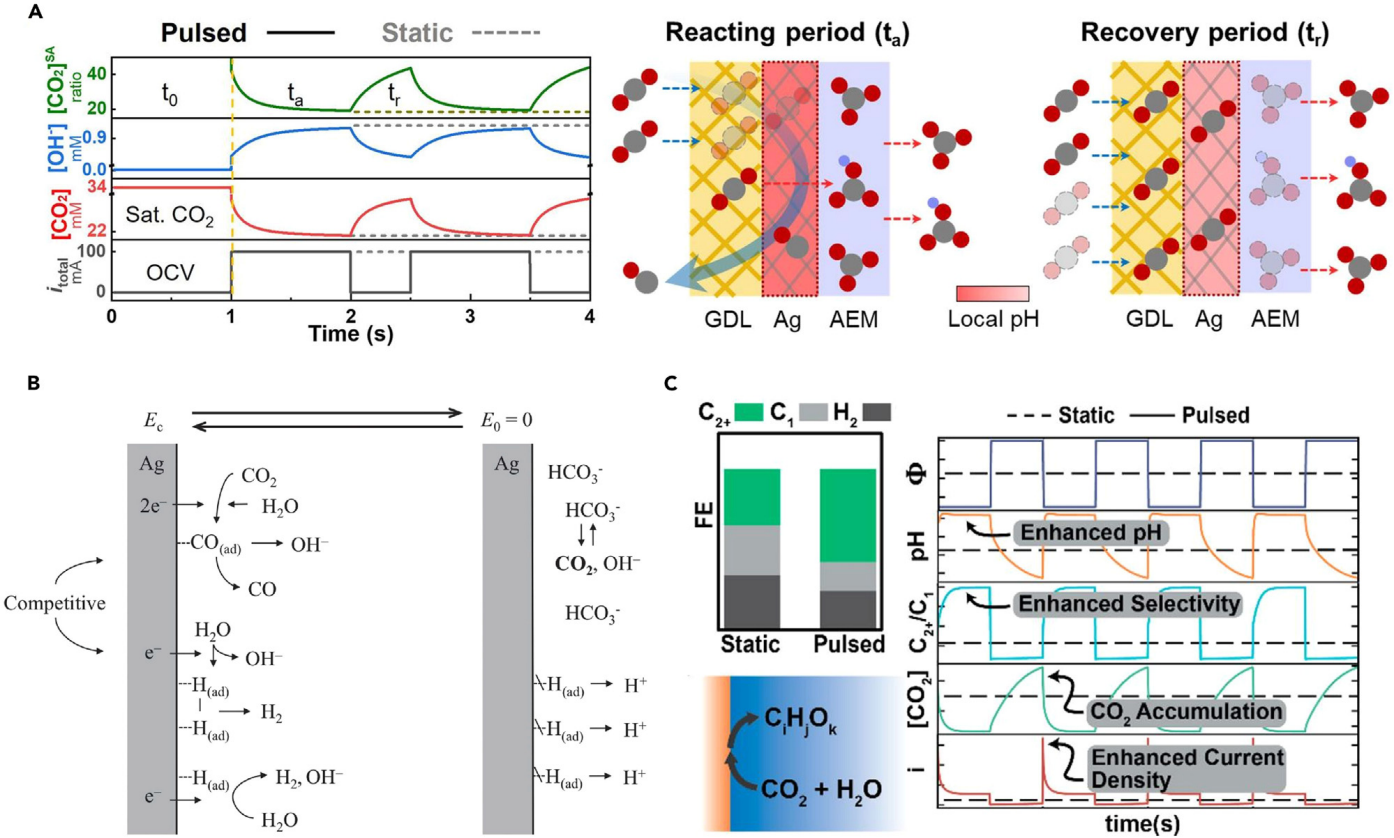
Effects of pulse-assisted electrolysis on mass transport (A) Modeling results of the local CO_2_, OH^−^, and [CO_2_]^SA^ concentration changes over time and illustrations of product pathways during the reacting and recovery periods (reprinted with permission from Xu et al.^55^ Copyright 2022, Angewandte Chemie International Edition). (B) The CO_2_RR mechanism on the surface during potential-pulse polarization (reprinted with permission from Oguma et al.^56^ Copyright 2020, ECSJ). (C) The FEs of CO_2_ reduction products (left) and CO_2_ concentration, partial current density, selectivity, pH changes over time (right) during static and pulsed methods (reprinted with permission from Bui et al.,^27^ Copyright 2021, American Chemical Society).

As the electrochemical reaction occurs on the electrode surface, gaseous CO_2_ reactants must be delivered to near the electrode surface to undergo electrochemical CO_2_ reduction catalysis. In this regard, effective mass transport of CO_2_ is crucial for electrochemical CO_2_RR. Xu et al.^55^ evaluated CO_2_RR performance with the surface-accessible CO_2_ concentrations ([CO_2_]^SA^), defined as the local [CO_2_]/[OH]^−^. The authors focused on the challenge faced in anion exchange membrane (AEM)-based CO_2_ electrolysis in which low local CO_2_ conversion rates occur in large current densities due to CO_2_ neutralization. To overcome these challenges, the authors developed three strategies: enhancement of the catalyst layer thickness, increased CO_2_ pressure, and the pulsed method. Mass-transport modeling results during the pulsed current method, which predicted dynamic changes to the local OH^−^ and CO_2_ concentrations near the catalyst surface compared to the static method, showed that a pulsed current can regulate the microenvironment (Figure 5A). When the pulse method was applied, during the t_a_, which represents the reacting period, local CO_2_ decreased while the OH^−^ concentration increased. During the recovery period, OH^−^ concentration decreases due to neutralization although CO_2_R does not occur. During the pulse operation, the OH^−^ concentration decreases during the t_a_ due to CO_2_ neutralization, resulting in higher [CO_2_]^SA^ compared to the static method. Oguma et al.^56^ employed the potential-pulse polarization method using 1-ethyl-3-methylimidazolium ethyl sulfate (EMISE) to improve the CO_2_RR performance. When a polarization potential is applied, CO_2_ is converted to CO, reducing the CO_2_ concentration and promoting the HER. The authors reported that applying the subsequent resting potential can induce the diffusion of the CO_3_^2−^ and HCO_3_^−^ species from the bulk solution to regenerate the CO_2_, as depicted in Figure 5B. Bui et al.^27^ attempted to reveal how the pulse method affects the microenvironment and the selectivity of products (Figure 5C). The authors developed a continuum model of CO_2_ reduction, which could provide the transient data of pH, CO_2_ concentrations, and overpotentials. Using this multiphysics model, the authors confirmed that the repetitive access to a transient state characterized by elevated pH and CO_2_ concentration under high cathodic overpotential led to an enhanced FE of C_2+_ species. They confirmed that high local CO_2_ concentration is quickly achieved within the first second, resulting in a significant increase in current density, which in turn quickly elevates the surface pH. This transient phenomenon observed in the pulse method enables for the increased current density and higher pH levels compared to static electrolysis. This time-dependent continuum model results provided insights into the role of microenvironment at the cathode.^26^ Additionally, Kim et al.^52^ investigated the role of pulsed electrolysis using a temporal product analysis based on DEMS data, showing the concentration changes of C_2_H_4_, H_2_, and CO near the cathode and the surface concentration of adsorbed CO. The authors proposed that pulsed electrolysis increases the local concentration of CO_2_, consequently elevating the selectivity of C_2_ products. In summary, pulses mitigate catalyst degradation by restructuring the surface or oxidizing absorbates. Previously reported studies claimed that the key point in mitigating poisoning is the restructuring of the surface. In Xu’s work, the 20-min paused period was implemented to mitigate salt formation by providing the time for protons to diffuse back to the interface and for the bicarbonate to leave the surface.^41^ Similarly, Xu’s research utilized simulations to confirm that pulses play a role in keeping carbonate ions away from the electrode.^42^ Indeed, some studies focus on oxidizing adsorbates to prevent catalyst degradation. In Friebe et al.’s work, the authors confirmed that anodic pulse oxidizes the adsorbed poisoning species, converting them into non-poisoning compounds.^23^ However, when considering the other effects of pulses discussed in different sections, such as surface reconstruction and adsorbate rearrangement, it appears that altering the surface structure environment can be another key factor in mitigating poisoning. We believe both explanations would be valid depending on the experimental conditions. To enhance understanding of the papers we reviewed, we have included a Table 1 summarizing the conditions implemented in each paper, categorized into four hypotheses.

**Table 1:** Summary of the applications of pulsed CO_2_ electrolysis

Catalyst	Pulse profile							
	Cell type	Electrolyte	Cathodic	Anodic	Stability	Selectivity	Effect	Reference
Cu	Flow-cell	4 M KCl	1.25 A cm^-^^2^ t_c_ = 9000 s	Pause t_a_ = 20 min	4 h	C_2_H_4_: ∼40% Current density: 470 mA cm^−2^	Pulsed current enhanced stability in bulk acidic media^(A)^	Xu et al.^41^
Cu, Cu_2_O, CuO	Flow-cell	1 M KOH	(1) Apply −2.2 V (vs. Ag/AgCl) for 1 h (2) hold at 1.5 or 0.1 V (vs. Ag/AgCl) for 5 min (3) scan from −1.1 V to −0.5 V (vs. Ag/AgCl) at 50 mV s^-1^ for 20 cycles		6 h	H_2_: ∼40% C_2_H_4_: ∼28%	Changes in the C_2_H_4_ activity and selectivity^(B)^	Lee et al.^57^
Cu/Ag alloy	H-cell	0.1 M KHCO_3_	−2.5 V ≤ E_c_ ≤ −1.9 V (vs. Ag/AgCl) t_c_ = 5 s	0.25 V ≤ E_a_ ≤ 0.9 V (vs. Ag/AgCl) t_a_ = 5 s	8 h	C_2_H_4_: ∼12.8%, C_2_H_5_OH: ∼17.3%, CH_3_CHO: ∼24.1%	C_2_ product selectivity increase^(C)^	Ishimaru et al.^51^
Cu	H-cell	0.1 M KHCO_3_	Galvanostatic reduction (−5 mA cm^−2^) with 3 CV scans (−1.2 to −0.1 V [vs. Ag/AgCl], 100 mV s^-1^) every 15 min, 1, 2, or 5 h or with potential steps to −0.25, −0.5, −1.2 V (vs. Ag/AgCl) for 84 s every 2 h		10 h	CH_4_: ∼50% CO: ∼50%	Selectivity of CO and CH_4_ varies depending on the step size of the CV scan^(C)^	Lim et al.^58^
Ag	H-cell	0.1 M KHCO_3_	$-2.5\;V\leq E_{c}\leq -1.75\;V$ (vs Ag / AgCl) t_c_ = 5 s	$-0.8\;V\leq E_{a}\leq -0.1\;V$ (vs Ag / AgCl) t_a_ = 5 s	10 h	CH_4_: ∼55% CO: ∼70%	Change in hydrocarbon selectivity^(C)^	Shiratsuchi and Nogami^50^
Cu_2_O nanocubes	Flow-cell	1 M KOH	−0.7V(vsRHE) t_c_ = 1 s	0.6V≤Ea≤1.5V(vsRHE) t_a_ = 1 s	10 h	CH_4_: ∼48.3% C_2_H_4_: ∼43.6% C_2_H_5_OH: ∼19.8%	At an E_a_ of 0.9 V, C_2_ is favored, while at 1.2 V, CH_4_ selectivity increased^(B)^	Jeon et al.^43^
Ag	MEA	0.1 M KHCO_3_	3.3V(fullcellvoltage) t_c_ = 1 s	2.0V(fullcellvoltage) t_a_≥ 0.5 s	13 h	CO: ∼70% Current density: 350 mA cm^−2^	Increased CO selectivity^(D)^	Xu et al.^55^
Cu-DHP	Flow-cell	0.1 M KHCO_3_	−1.38V(vsAg/AgCl) t_c_ = 25 s	−1V(vsAg/AgCl) t_a_ = 5 s	16 h	C_2_H_4_: ∼23%	Stable C_2_H_4_ formation^(B)^	Jannsch et al.^59^
Pb foil	Flow-cell	0.1 M KHCO_3_	−1.1 V (vs. RHE) t_c_ = 95 s	0.1 V (vs. RHE) t_a_ = 5 s	16 h	HCOO^−^: ∼48%	High selectivity for formate^(B)^	Stamatelos et al.^60^
Cu	H-cell	KHCO_3_ and KOH at varying concentrations	−1.05V(vsRHE) t_c_ = 50 ms	0.4V(vsRHE) t_a_ = 50 ms	24 h	In KHCO_3_ CH_4_: ∼40%, CO: ∼70% In KCl C_2_: ∼71%	Variation in selectivity with electrolyte concentration^(C)^	Casebolt et al.^61^
Cu foil	H-cell	0.1 M KHCO_3_+0.1 M K_2_BDC	−1.0V(vsRHE) t_c_ = 50 s	1.25V(vsRHE) t_a_ = 3 s	25 h	C_2+_: ∼70.3%	Achieved high selectivity for C_2+_ products. Identified the catalysts’ optimal oxidation state^(B)^	Xu et al.^47^
Cu foil	H-cell	0.1 M KHCO_3_+0.1 M KI	−1.2V(vsRHE) t_c_ = 50 s	0.4V(vsRHE) t_a_ = 2 s	36 h	C_2+_: ∼81.2%	High C_2+_ selectivity. The surface structure and oxidation state of Cu could be recovered periodically by the pulse^(B)^	Xu et al.^16^
Cu	Flow-cell	2.5 M KHCO_3_+0.5 M K_2_CO_3_	−1.2V(vsRHE) t_c_ = 15 min	Off t_a_ = 15 min	36 h	C_2_H_4_: ∼40% Current density: 1 A cm^−2^	Achieved high current density and C_2_H_4_ selectivity^(B)^	Nguyen et al.^62^
Pb_80_Ag_20_/C	H-cell	0.5 M NaHCO_3_	−0.8V(vsRHE) t_c_ = 590 s	1.22V(vsRHE) t_a_ = 10 s	45 h	HCOO^−^: ∼97.8%	Prevented surface poisoning through a cyclic two-step process^(A)^	Lee et al.^63^
Cu	H-cell	0.1 M NaHCO_3_ and 0.5 M KHCO_3_	3 scans every 5 min from −1.72 to +1.3 V (vs. NHE) at 5 V s^-1^		50 h	CH_4_: ∼10% C_2_H_4_: ∼25%	Maintained high hydrocarbon selectivity over a long period^(B)^	Jermann and Augustynski^64^
Cu-DHP	H-cell	0.1 M KHCO_3_	$-1.8\;V\leq E_{c}\leq -1.5\;V$ (vs Ag / AgCl) t_c_ = 5–500 s	$-0.88\;V\leq E_{a}\leq 0.15$ V (vs Ag / AgCl), t_a_ = 5 s	95 h	CH_4_: ∼50% C_2_H_4_: ∼35%	HER suppression and stable hydrocarbon formation^(B)^	Engelbrecht et al.^26^
ZnO nanorods	MEA	1 M KOH	−3.6 V for 20 min; then CV at −2 V to 1 V		100 h	CO: ∼83% Current density: 160 mA cm^−2^	High CO selectivity and stability^(B)^	Stamatelos et al.^65^
Cu	Flow-cell	1.0 KHCO_3_	150 mA cm^−2^ t_c_ = 45 min	1.0 mA cm^−2^ t_a_ = 24 s	200 h	C_2_H_4_: ∼40% Current density: 150 mA cm^−2^	Achieved high stability through the application of pulsed current^(A)^	Obasanjo et al.^15^
Cu and Ag	MEA	0.1 M KHCO_3_	−3.6V(fullcellvoltage) t_c_ = 60 s	−2V(fullcellvoltage) t_a_ = 30 s	236 h	In Ag CO: ∼90% Current density: 170 mA cm^−2^ In Cu C_2+_: ∼80% Current density: 138 mA cm^−2^	Increased stability due to the inhibition of salt precipitation^(A)^	Xu et al.^42^
Cu_3_(DMPz)_3_	H-cell	0.1 M KCl	ALPs-1 −1.28V(vsRHE) t_c_ = 300 s ALPs-2 0.58V(vsRHE) −1.08V(vsRHE) t_c_ = 30 s, 600 s	ALPs-1 1.27V(vsRHE) t_a_ = 30 s ALPs-2 0.42V(vsRHE) t_a_ = 30 s	In ALPs-1: 300 h In ALPs-2: 145 h	In ALPs-1 CH_4_: ∼80.3% In ALPs-2 C_2_H_4_: ∼70.7%	Selective tuning and enhanced stability based on pulse profile^(B)^	Zhang et al.^18^
NiO-8YSZ	Solid oxide electrolytic cells (SOEC)	8YSZ	Pulsed current range of −100 to −300 mA cm^-^^2^ sinuous amplitude: 10 mV		800 h	CO_2_ conversion rate: 52%	High stability through pulsed current^(A)^	Wu et al.^66^

The effects of pulsed electrolysis are classified into following categories: (A) inhibition of catalyst poisoning, (B) surface reconstruction, (C) surface coverage rearrangement, and (D) mass transport limitation mitigation.

## Shape of the pulsed electrolysis

**Figure 6:**
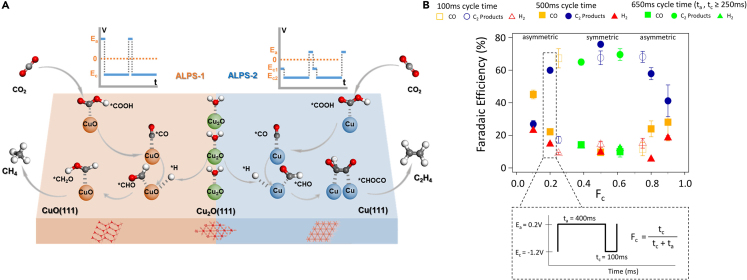
Shape of pulse profile (A) The CO_2_RR mechanisms of CH_4_ and C_2_H_4_ production on the catalyst surface using the ALPs-1 and ALPs-2 methods, respectively (reprinted with permission from Zhang et al.^18^ Copyright 2023, American Chemical Society). (B) Product selectivity with different pulse time. Each point is an average over the 2 h experiment with error bars representing one standard deviation from the mean. (reprinted with permission from DiDomenico et al.^17^ Copyright 2021, American Chemical Society).

So far, we have discussed various strategies for modulating product selectivity by changing the pulse profile through E_c_, E_a_, t_a_, and t_c_. In recent studies, emphasis has shifted toward exploring pulse symmetry. Zhang et al.^18^ introduced an asymmetric low-frequency pulsed strategy (ALPs) capable of sustained CH_4_ and C_2_H_4_ production over 100 h. To achieve the high selectivity, anodic potentials of two ALPs methods, ALPs-1 and ALPs-2, were selected based on the anodic peaks of Cu_3_(DMPz)_3_. Prior studies have shown that product selectivity varies depending on Cu species.^16^^,^^46^^,^^67^ The authors identified the different Cu species through microscopy analysis, revealing the effects of the ALPs methods. In addition, when increasing the electrolysis time, aggregation traditionally occurs, decreasing the catalyst activity. Optimal asymmetrical adjustments to the applied anodic potential time were employed to sustain effective catalytic activity. Based on the experimental observations, the authors proposed two distinct ALPs methods respectively focusing on generating CH_4_ or C_2_H_4_ products (Figure 6A).

In contrast, DiDomenico et al.^17^ reported that a symmetric pulse sequence is better for producing C_2_ products (Figure 6B). The authors investigated pulse characteristics, including applied potential, time, and pulse symmetry, for optimizing C_2_ formation conditions. Notably, the effect of pulse symmetry on the overall reaction rate was posited to surpassing the combined effects of surface reaction and desorption energy, as reported by Shetty et al.^68^ The authors attempted to reveal the correlation between symmetric pulse shape and C_2_ selectivity for the first time and suggested that pulse shape can regulate adsorbate coverage to achieve high C_2_ selectivity.

## Improvements in catalytic stability

For decades, Cu-based catalysts have been widely studied for CO_2_ reduction catalysis, as only copper-based compounds are known to be able to produce C_2__+_ products. From an industrial perspective, it is important to find a catalyst with high selectivity and long-term stability. While Cu-based electrocatalyst exhibit superb selectivity, the catalytic stability has not been good enough. Poor stability is normally the result of salt formation, catalyst degradation, poisoning, flooding, and undesirable surface reconstruction. Therefore, ensuring long-term stability for CO_2_RRs is an important objective. Recent studies showed that pulse-assisted electrolysis can address these issues.

Obasanjo et al.^15^ reached 150 mA cm^−2^ for over 60 h and maintained a minimum FE of 40% for C_2_H_4_ with an *in situ* catalyst regeneration strategy. Alternating current regenerated the Cu surface, which enhanced the catalyst’s lifetime. They also confirmed using SEM imagery of the catalyst surface that during the alternating current cycle, Cu species migrated from the polytetrafluoroethylene (PTFE) substrate, resulting in catalyst degradation.

**Figure 7:**
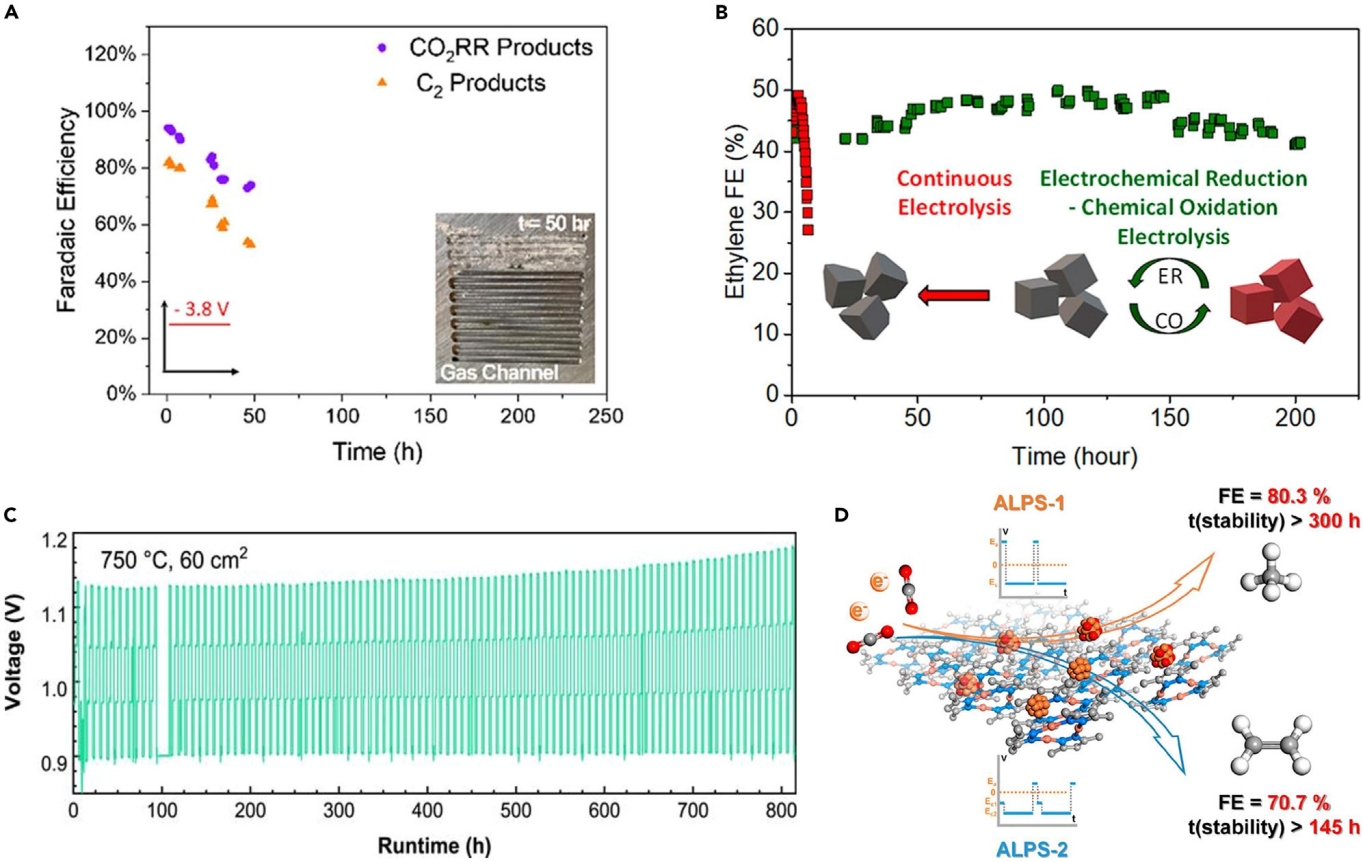
Pulsed CO_2_ electrolysis stability (A) Selectivity of CO_2_RR products during the long-term operation (reprinted with permission from Xu et al.^42^ Copyright 2021, American Chemical Society). (B) A 40% FE of C_2_H_4_ for 200 h using electrochemical reduction-chemical oxidation electrolysis (reprinted with permission from Nguyen et al.^62^ Copyright 2022, American Chemical Society). (C) Long-term stability data under pulsed current operation (reprinted with permission from Wu et al.^66^ Copyright 2022, Wenzhou University and John Wiley & Sons Australia, Ltd). (D) The FE and stability data for two ALPs methods (reprinted with permission from Zhang et al.^18^ Copyright 2023, American Chemical Society).

Xu et al.^42^ proposed a self-cleaning CO_2_ reduction system that prevents salt formation and increases the operating time to 236 h while achieving an 80% C_2_ selectivity (Figure 7A). Nguyen et al.^62^ reported “on” and “off” strategies that can achieve over 40% FE for C_2_H_4_ for 200 h at 150 mA cm^−2^ in a neutral pH electrolyte (Figure 7B). They combined electrochemical reduction and chemical oxidation, each part proceeding with 15-min time intervals. The catalyst was regenerated during the “off” time step with slow chemical oxidation. As the “on” times increased, current density also increased, which finally resulted in an unrecoverable catalyst. Wu et al.^66^ used solid oxide electrolytic cells (SOECs) for CO_2_RRs that aim for energy storage. Using this system under the pulsed current method proceeded for 800 h with a 52% conversion rate of CO_2_ (Figure 7C). Zhang et al.^18^ developed ALPS methods, which can achieve high selectivity and stability for CH_4_ (80.3% FE for 300 h) or C_2_H_4_ (70.7% FE for 145 h), as mentioned in section shape of the pulsed electrolysis (Figure 7D).

## Computational modeling of pulsed CO_2_ electrolysis

Recent research on pulsed electrolysis has contributed to advancements in our understanding of the mechanisms underlying pulsed CO_2_ electrolysis through *in situ* time-resolved analysis.^46^^,^^69^ While there are still unresolved questions, computational modeling can help clarify these mechanisms. Particularly, since it is challenging to analyze the solid-liquid interface using spectroscopic approaches, quantum mechanical methods offer pathways to investigate this interface,^70^ allowing us to understand the impact of various parameters on electrolyzer performance and enabling efficient simulations for the optimization of the electrolyzer system. The pulse profile, characterized by E_a_, E_c_, t_a_, and t_c_, has a considerable number of possible variations, and even slight differences can lead to varying results. Conducting experiments to explore all these possibilities would be time-consuming and costly. Therefore, it is necessary to simulate various pulse profiles through modeling to find the optimal conditions. In this section, we summarize recent studies on pulsed electrolyzer modeling, outline the limitations of the current models, and suggest specific research avenues necessary to overcome these limitations.

### Continuum scale multiphysics modeling

In pulsed electrolysis, the pulse profile can create differences in mass transport limitations, surface reconstruction, and surface coverage rearrangement. Pulsed electrolysis can accumulate higher concentrations of CO_2_ on the catalyst surface due to the CO_2_ replenishing effect when applying longer duration anodic potentials.^55^ The changes in mass transport induced by pulse application and the resulting alterations in surface reaction kinetics at the continuum scale can be simulated by reaction-diffusion models using the Nernst–Planck equation and the Butler–Volmer equation.^71^

Equation 3 is the Nernst–Planck equation, which can describe the transport of species in an electrochemical system. The first term corresponds to diffusion driven by the concentration gradient, the second term indicates electromigration due to the electrical potential gradient, and the third term denotes convection induced by fluid velocity.^72^ When applying a constant voltage, the diffusion and migration of reactants (CO_2_, H_2_O, etc.), products, and charge states (electrolyte cation, (bi)carbonate, etc.) can exhibit different transport characteristics than when applying a pulsed voltage. For example, when using a membrane electrode assembly (MEA) with an AEM, applying a more positive anodic potential can inhibit the migration of cations from the anolyte to the cathode due to electro-osmotic drag, preventing salt precipitation.^72^ Therefore, changes in the electrical potential gradient and the concentration gradient induced by the application of pulsed voltage can influence species transport.

(Equation 3) $$J\left(x\right)=-D\nabla c+\frac{Dze}{k_{B}T}cE+cv$$

Equation 4 represents the Butler–Volmer equation, describing the partial current densities of individual products at the catalyst surface, which correspond to the reaction rates. The kinetics equations assume a first-order dependence of the partial current densities of CO_2_ reduction products on the local CO_2_ concentration.^27^ In this equation, the variables that change with pulse application include the local CO_2_ concentration ($c_{{\text{CO}}_{2}}$), which varies due to the CO_2_ replenishing effect, and the overpotential (*η*), which changes according to the pulse profile. This equation poses challenges in capturing complex catalytic surface reactions. Therefore, fitting kinetic parameters based on experimental results such as polarization curves and product distributions allows for more accurate modeling. The most commonly used fitted parameters include the exchange current density ($i_{o,k}$) and transfer coefficient ($\alpha_{c,k}$), both of which vary depending on the pulse profile.^71^

(Equation 4) $$i_{k}=-i_{o,k}{\left(\frac{c_{{\text{CO}}_{2}}}{c_{\text{ref}}}\right)}^{\gamma_{{\text{CO}}_{2,k}}}\;\exp\;\left(-\frac{\alpha_{c,k}F}{RT}\eta_{c,k}\right)$$

This continuum scale model faces challenges in expressing the changing current-potential relationship due to the complex electric double-layer structure at the electrode-electrolyte interface. Moreover, it assumes a low adsorbate coverage on the electrode surface, and when the surface adsorbate coverage is high, more complex expressions may be required.^71^ Nevertheless, using this model, one can simulate the changes in mass transport within the boundary layer induced by pulsed methods, thereby capturing variations in the FE of the products.^27^ Furthermore, by modeling the effects of pulsing on local pH and potential gradient changes, one can simulate the influence on carbonate generation and migration.^42^ The Butler–Volmer kinetics and buffer reactions can account for changes in local CO_2_ concentration and pH resulting from pulsing.^27^ When modeling the mass transport layer at the continuum scale in response to a pulse profile, it is possible to simulate (1) changes in species transport, (2) the influence of CO_2_ buffer reactions, and (3) the impact of reaction kinetics due to changes in local CO_2_ concentrations and applied potentials. In the following sections, we introduce the utilization of this continuum-scale model in research involving pulsed electrolysis.

**Figure 8:**
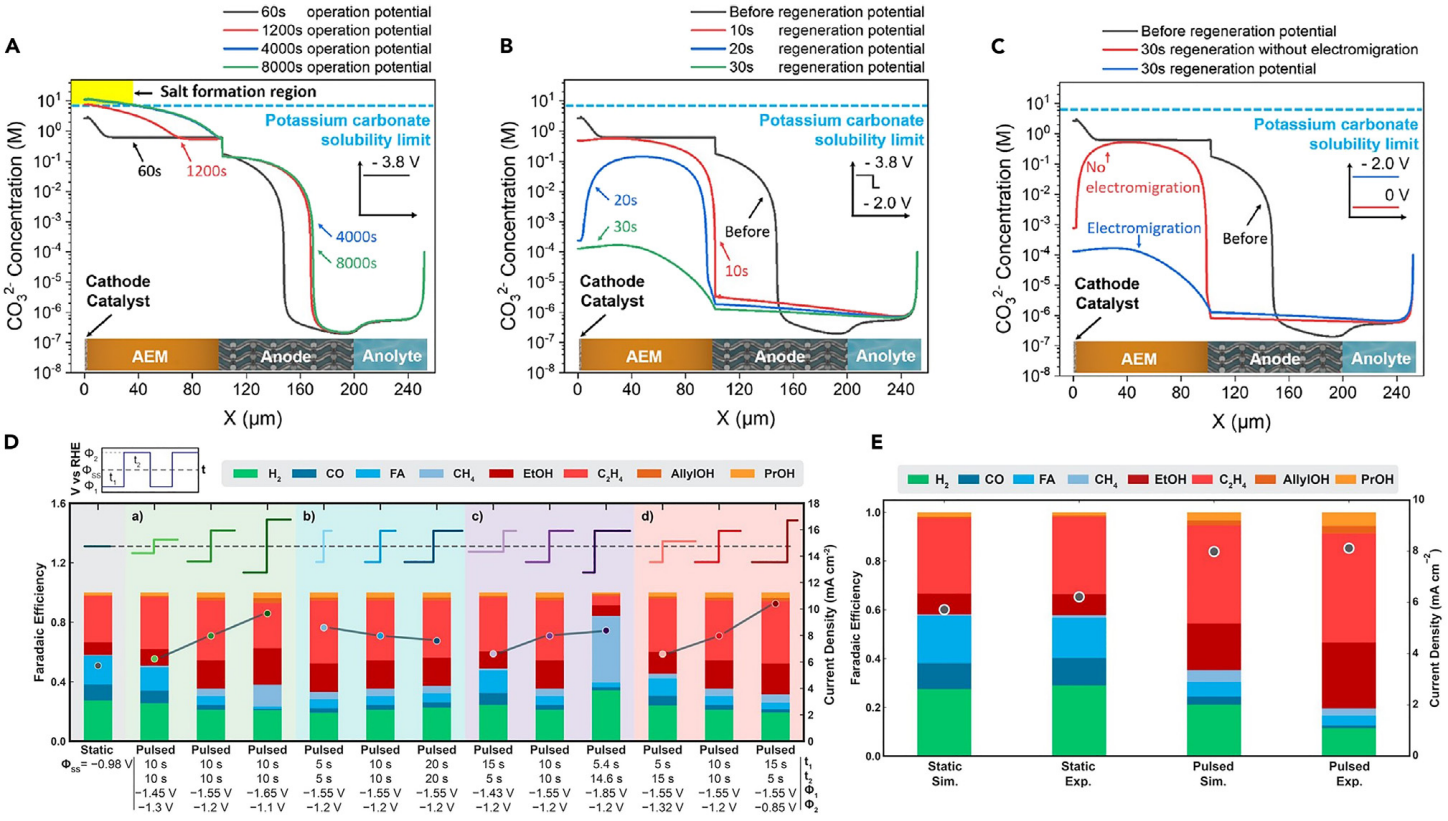
Continuum scale multiphysics model simulations of pulsed CO_2_ electrolysis Carbonate concentrations within the MEA. (A) Different operational times for continuous operation at −3.8 V (current density: 172 mA cm^-^^2^), (B) different regeneration times (regeneration voltage = −2.0 V) after 60 s of operation at −3.8 V, and (C) a comparison of electromigrative (blue) and concentration-driven (red) diffusive effects (reprinted with permission from Xu et al.^42^ Copyright, American Chemical Society 2021). (D) Effect of pulse characteristics on the FE (stacked bars) of products and total current density (dots and lines). (E) A comparison of the FEs (stacked bars) of products and the total current densities (dots) under static and pulsed potentials with simulated (Sim.) experimentally observed (Exp.) results (reprinted with permission from Bui et al.^27^ Copyright American, Chemical Society 2021).

Kimura et al.^24^ used Gupta’s diffusion model to understand changes in the concentrations of reactants and products near the electrode surface. This presents a mathematical model that reflects the role of CO_2_ reactants and buffers through electrode–electrolyte boundary layer modeling. Also, it can successfully simulate changes in local conditions around the copper electrode when a pulse profile is applied.^73^ Here, the authors calculated the variation in CO_2_ concentration based on the length of the pulses (t_a_, t_c_) through modeling. Kim et al.^52^ simulated the major physical processes occurring in the boundary layer to explain changes in product distribution during pulsed electrolysis. Through this model, it becomes possible to simulate (1) changes in local CO_2_ concentrations due to the CO_2_ replenishing effect and (2) the resulting variations in local pH. Therefore, it is possible to simulate the resolution of mass transport limitations in response to the pulse profile. Xu et al.^42^ simulated pulsed electrolysis using a relatively complex MEA electrolyzer model. Solid salts are formed when accumulated carbonate ions generated by the buffer reaction of CO_2_ exceed their solubility limit, leading to their precipitation by cations and carbonates.^74^ This model simulated (1) a decrease in OH^−^ generation and a reduction in carbonate production around the cathode during the application of the regeneration potential, as depicted in, Figures 8A and 8B and (2) an increase in carbonate concentrations around the cathode due to a weakening of the electromigration force resulting from the application of a more positive anodic potential, as illustrated in Figure 8C. As a result, the authors identified an appropriate pulse profile to prevent salt precipitation, and by applying this in experiments, they enhanced the stability of the electrolyzer.

Until now, pulse modeling has primarily been used for mechanistic interpretation. In contrast, Bui et al.^27^ conducted research on identifying the ideal pulse profile using the multiphysics model (Figure 8D). Based on the study by Kim et al.,^52^ they applied potentials within the range where surface rearrangement and oxidation do not occur. This model simulated (1) the cathode mass transport boundary layer of the H-cell and (2) reflected the transient effects of local CO_2_ concentrations, pH, and overpotentials on the reaction kinetics of individual product formation. The Butler–Volmer equation, used to calculate the partial current density of products, derives reaction kinetics under relatively simple assumptions.^71^ To improve consistency between experimental data and simulation results, parameter fitting for the exchange current density (*i*_o,k_) and cathodic transfer coefficient (*α*_c,k_) was conducted. The simulation results generally align with the experimental results (Figure 8E), but when compared to the static case, some discrepancies are evident in the pulse electrolysis simulation. In this model,^27^ the authors explained that an increase in the local CO_2_ concentration leads to an increase in partial current densities of CO_2_-derived products, whereas an increase in pH reduces the current densities of C_1_ and H_2_ products relative to C_2__+_ products.^75^^,^^76^ However, the assumption of a low coverage of adsorbed CO, which applies during static electrolysis, may not hold true during the transient high CO_2_ concentrations and current densities of pulsed electrolysis.^27^^,^^77^ The authors suggested that, in pulsed electrolysis, the suppression of H_2_ formation due to a higher coverage of adsorbed CO can explain the higher H_2_ FE observed in the simulation. To capture these effects, a complete microkinetic model is necessary.

The Butler–Volmer equation is based on the simplifying assumption that the reaction is controlled by a particular rate-determining step. However, most electrochemical reactions involve multiple steps, and the rate-determining step can vary depending on the operating potential and local reaction conditions. Additionally, as all reactions occur at the same catalyst sites, competition among different species can influence the adsorption of different reactants and intermediate species onto the catalyst surface.^78^ To simulate the changes in surface reactions due to the varying local environment in pulsed electrolysis, microkinetic models are necessary. Microkinetic modeling can be employed using density functional theory (DFT)-derived parameters to explain and predict the catalytic activity for various electrochemical reactions.^71^

### Surface reaction mechanism modeling

Bui et al.^27^ simulated the impact of fluctuations in mass transport limitations due to a pulsed potential on reaction kinetics by applying voltages in a model where surface reconstruction and oxidation do not occur. Several studies have argued that mass transport limitations are not a primary factor influencing selectivity changes.^24^ In pulsed electrochemical systems, product selectivity can vary based on changes in the structure and oxidation state of the electrode surface.^79^ Additionally, the abrupt switching of the applied potential can impact the arrangement of the electric double layer and the coverage of surface adsorbates.^25^^,^^27^ Therefore, to implement a more accurate model across all potential ranges, it is necessary to incorporate the impact of surface reconstruction and surface coverage rearrangement resulting from the pulse profile. With our current knowledge, understanding the reaction mechanisms behind these phenomena that are modified by the pulse profile remains a challenge. Therefore, to bridge the knowledge gap, one can investigate the reaction mechanism of pulsed electrolysis through experiments and DFT calculations. In this section, we introduce research using computational methods to predict phenomena occurring on the catalyst surface in pulsed CO_2_ electrolysis.

**Figure 9:**
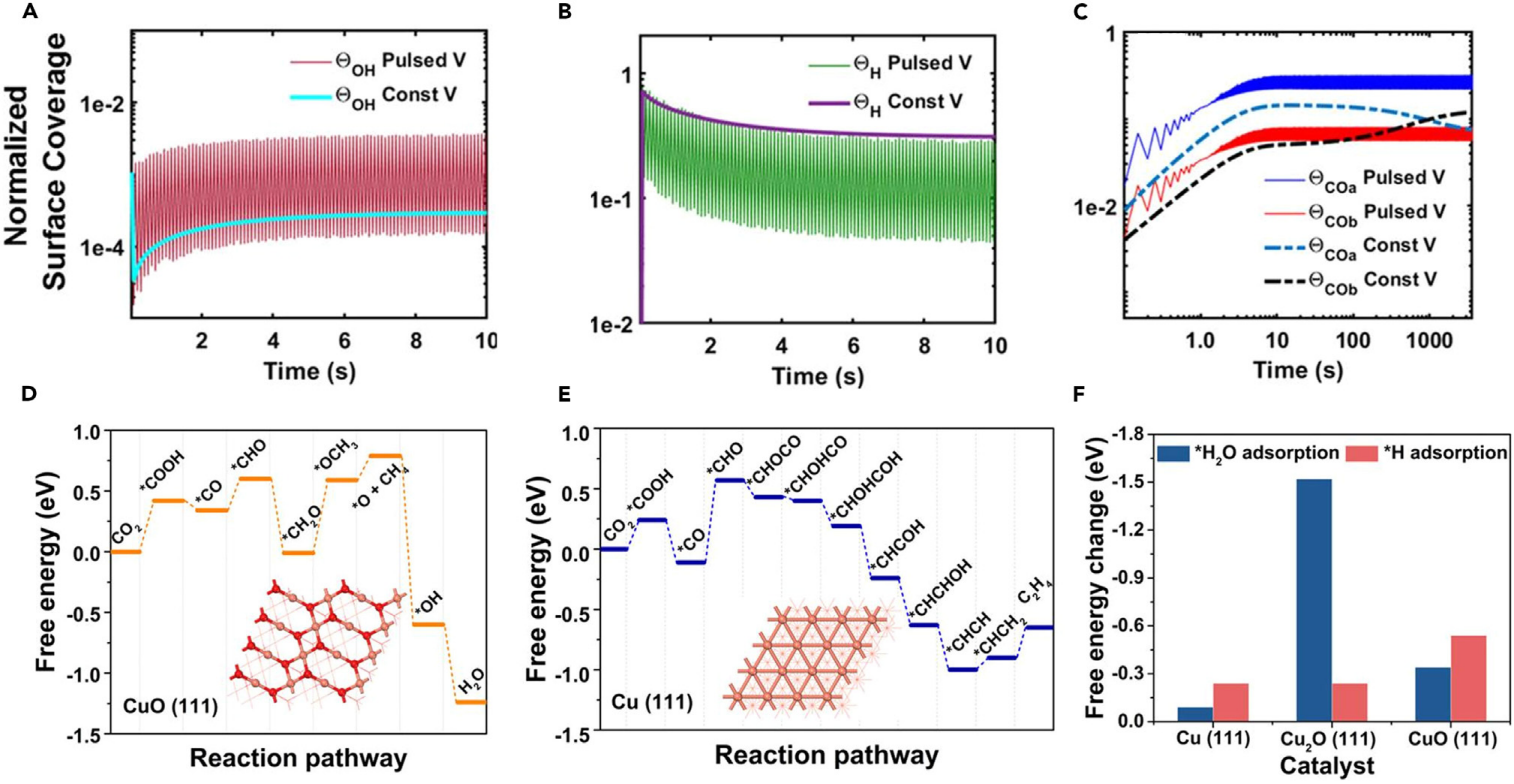
Pulsed CO_2_ electrolysis surface coverage variation Relative surface coverage under pulsed and constant potentials of (A) adsorbed OH over 10 s, (B) adsorbed H over 10 s, and (C) adsorbed CO_atop_ (COa) and CO_bridge_ (COb) over 1 h. The pulse profile for these models was t_a_ = t_c_ = 50 ms, and the models assumed a competitive quaternary Langmuir isotherm (reprinted with permission from Kimura et al.^25^ Copyright 2020, American Chemical Society). Gibbs free energy profiles for (D) CH_4_ production on the CuO (111) plane with ALPs-1 and (E) C_2_H_4_ production on the Cu (111) plane with ALPs-2. (F) Change in Gibbs energy for ∗H_2_O and ∗H adsorption on the Cu (111), Cu2O (111), and CuO (111) planes (reprinted with permission from Zhang et al.^18^ Copyright 2023, American Chemical Society).

In the mechanisms of pulsed electrolysis, changes in the surface coverage of hydrogen (H_ads_) and hydroxide (OH_ads_) are known to play a significant role.^49^^,^^50^ Iijima et al.^80^ suggested that the increased surface coverage of OH_ads_ induced by pulsed conditions promotes a near-neighbor coupling effect with CO_atop_, preventing the generation of CO_bridge_ and suppressing the HER. Kimura et al.^25^ modeled surface adsorption on a copper surface under pulsed potential for a more precise mechanistic analysis. This model was developed based on the diffusion layer transport model of Gupta et al.^73^ and considered the adsorption phenomena using a competitive Langmuir isotherm with four components: H_ads_, OH_ads_, CO_atop_, and CO_bridge_.^24^^,^^73^^,^^80^ It calculated the slow transfer reaction from CO_atop_ to CO_bridge_, assuming a first-order reaction rate, only when OH^−^ was minimally present. However, determining the exact values of adsorption constants (k_ads,i_) in pulsed potential systems can be challenging. Therefore, the model illustrated how pulsed and constant electrolysis dynamically vary by comparing their relative surface coverages.^73^^,^^81^ Figures 9A and 9B present the calculated variations in surface coverage of OH_ads_ and H_ads_, respectively, under the application of constant and pulsed voltages. Figure 9C illustrates the differences in the formation of CO_atop_ and CO_bridge_ under the same conditions. Ultimately, this model predicted that when the anodic potential is above +0.2 V, the electrode surface will repel H^+^ and promote the adsorption of OH^−^, preventing CO_bridge_ formation through near-neighbor coupling interactions with CO_ads_.^25^

Zhang et al.^18^ conducted DFT calculations to elucidate the CO_2_RR pathways in two pulse profiles with distinct asymmetric characteristics (ALPS-1, ALPS-2), aiming to understand the fundamental reasons behind their high respective selectivity for CH_4_ and C_2_H_4_. Based on high-resolution transmission electron microscopy (HRTEM), CuO (111) planes were used as models to investigate the reaction mechanism for CH_4_, and Cu (111) planes were similarly used for C_2_H_4_. As shown in the Figure 9D, the rate-limiting step in the CH_4_ production pathway appears to be between ∗CH_2_O and ∗OCH_3_. On the other hand, the C_2_H_4_ production pathway is as shown in Figure 9E, with ∗CO to ∗CHO as the rate-limiting step. These DFT calculations were experimentally identified by *in situ* attenuated total reflectance Fourier transform infrared spectroscopy (*in situ* ATR-FTIR). Additionally, to understand the role of the Cu_2_O (111) plane in both methods, the Gibbs free energy changes of ∗H_2_O and ∗H were calculated (Figure 9F). The Cu_2_O (111) plane showed high performance in ∗H_2_O adsorption, promoting hydrogenation in both methods. These computational results corroborated the experimentally observed mechanisms, representing the overall catalytic mechanisms of CH_4_ and C_2_H_4_ production, as they are shown in the Figure 6A.

Wu et al.^19^ increased the CO_2_-to-ethanol FE by 46.3% using an se-Cu_2_O/Ag catalyst under pulsed conditions. The repetitive regeneration of Cu^+^ species due to the pulsed oxidation potential was confirmed by *in situ* XAS and Raman spectroscopy. To clearly understand the variation in selectivity due to the presence of Cu^+^, DFT calculations were conducted for the hydrogenation and dehydrogenation of the ∗HCCOH intermediate in Cu/Ag (static case) and se-Cu_2_O/Ag (pulsed case). On a surface where stable Cu+ species are present (pulsed case), the formation energy of ∗HCCHOH (a precursor to ethanol) is reduced, and the hydrogenation of HCCOH is favored on se-Cu_2_O/Ag. These results demonstrated that under pulsed conditions, se-Cu_2_O/Ag exhibits an increased selectivity toward ethanol.

Integrating experimental results with DFT calculations can aid in elucidating the reaction mechanisms underlying the varying product selectivity during pulsed CO_2_ electrolysis. Pulsed electrolysis is characterized by dynamic surface changes (oxidation state, adsorbate coverage, etc.) induced by alternating anodic and cathodic potentials.^25^^,^^47^^,^^69^^,^^82^ However, the previous studies were unable to account for the dynamic changes in the catalyst surface itself.^18^^,^^19^ In other words, the research conducted DFT calculations based on information obtained experimentally from a fixed surface under pulsed conditions. Therefore, there is a limitation in capturing the real-time impact of the dynamically changing surface on reaction kinetics. If there is enough information about the reaction pathways and rate equations that vary with the pulse profile for pulsed-electrolysis CO_2_ reduction, a microkinetic model can be implemented. However, at the current state of research on this subject, the information on how the energy landscape changes according to the pulse is insufficient. Additionally, surface reaction kinetics can vary depending on the structure of the electric double layer (EDL). The EDL refers to the interface between the electrode and electrolyte, and it is influenced by factors such as the type of electrode, electrolyte conditions (pH, concentration, etc.), and the applied potential.^83^ However, there is a lack of research on the factors influencing changes in the microenvironment of the EDL under pulsed conditions.^84^

To implement more sophisticated pulsed CO_2_ electrolysis modeling, it is necessary to (1) dynamically calculate changes in the reaction mechanisms on the surface based on to the pulse profile, overcoming the challenges of non-steady-state surface DFT calculations and gaining a mechanistic understanding, and (2) understand the EDL structure that influences surface reactions under the pulsed potential, which is crucial for a comprehensive understanding of the factors affecting the surface reactions. If theoretical knowledge regarding pulsed CO_2_ electrolysis advances, the integration of microkinetic models into multiscale continuum models of the electric double layer and mass transport boundary layer would be possible. This integrative modeling would allow for more robust simulations.^85^ If a highly accurate model can be implemented, it becomes possible to simulate various pulse profiles, enabling efficient exploration of optimal conditions. Furthermore, by combining channel-scale models with process-scale models, more effective economic analyses can be conducted.^86^

## Economic analysis of the industrial scale pulsed CO_2_ electrolysis process

**Figure 10:**
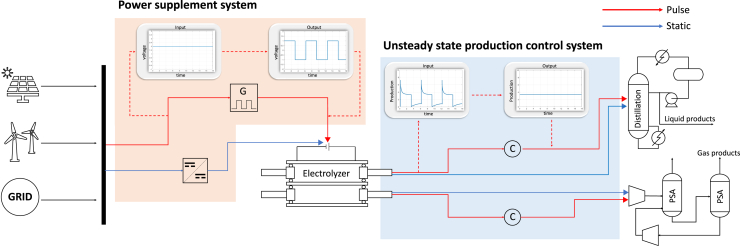
Industrial scale pulsed CO_2_ electrolysis process Schematic diagrams comparing CO_2_ electrolysis processes under constant (blue lines) and pulsed conditions (red lines).

To become a price-competitive CO_2_ electrolyzer, several key requirements need to be met, including high current density (>200 mA cm^−2^), high selectivity, and long-term operational capability (>8000 h or 1 year).^87^ Pulsed CO_2_ electrolysis processes have shown the potential to achieve higher stability and selectivity compared to constant processes. Furthermore, recent MEA-cell-based studies of pulse systems have demonstrated their ability to achieve higher current densities.^41^^,^^62^ However, unlike constant processes, pulsed electrolysis introduces additional considerations when assessing their economic viability (Figure 10). Therefore, confirming the feasibility and cost-effectiveness of implementing this process at an industrial scale is crucial. At the electrolyzer scale, optimizing the pulse profile is simply aimed at achieving high product selectivity and catalyst lifetime. When expanding to the process scale and optimizing the pulse profile, it is essential to consider the trade-offs between production loss, increased product selectivity, and catalyst lifetime. However, current research primarily focuses on optimizing for improved selectivity and stability, and this section will summarize the additional factors to consider when applying pulsed CO_2_ electrolysis at the process scale and their economic impacts.

### Separation and catalyst replacement costs

The CO_2_ electrolysis process requires the separation of the products generated in the electrolyzer. Typically, the gas product is separated using pressure swing adsorption (PSA), whereas the liquid product is separated through extraction or distillation.^88^^,^^89^ These downstream processes are crucial for enhancing the purity of the products. The costs associated with the separation process depend on factors such as product selectivity and the types of cogenerated substances (e.g., is it an azeotropic mixture?).^90^ If downstream separation processes are carried out without controlling selectivity, significant energy consumption is inevitable, leading to decreased economic viability. Pulsed electrolysis effectively suppresses hydrogen evolution reactions and enhances the selectivity of C_2+_ products, leading to a reduction in product separation costs.^17^^,^^52^^,^^69^ However, downstream processes involve the capture of unreacted and crossover CO_2_, which require substantial energy and result in high production costs.^90^ Furthermore, when the pulse is off, continuous CO_2_ injection can lead to a reduced fraction of CO_2_ being transformed into products. This leads to increased CO_2_ capture costs as the duty cycle decreases. The duty cycle is expressed as the ratio of the cathodic potential length applied to the total operation time. The increase in capture cost offsets the decrease in product separation cost, and this trade-off relationship varies depending on the product. Thus, when optimizing the pulse profile, it is important to consider not only selectivity but also variations in CO_2_ conversion to reduce the overall costs of downstream process.

For the stable operation of the process, it is necessary to replace the catalyst when degradation occurs. As the durability of the catalyst increases, the replacement cost can decrease.^6^^,^^91^ Catalyst degradation is a critical issue, as it leads to a decline in the quality of the product, necessitating the replacement of the catalyst. Moreover, catalyst replacement is accompanied by the drawback of production interruption, resulting in decreased productivity.^92^ Therefore, extending the replacement cycle is crucial for reducing replacement costs and enhancing overall process productivity. As mentioned in section improvements in catalytic stability, it is clear that pulsed electrolysis can attain a notably elevated level of stability.

### Process operation and power supplement system

The conventional method requires disassembling the electrolyzer and modifying its intrinsic characteristics to control performance.^93^ In contrast, pulsed electrolysis offers the advantage of more flexible operation by simply adjusting the applied voltage. However, pulsed electrolysis results in unsteady-state product generation from the electrolyzer. Consequently, it is necessary to control the unsteady-state production by implementing effective strategies to ensure consistent production rates. Since downstream processes in the overall electrolysis process, like separation, must operate under constant conditions,^90^ additional control is needed for this dynamic system. For example, in the green ammonia synthesis process, the large variability in H_2_ production due to fluctuating renewable energy usage results in unsteady H_2_ production. To control this variability, a consistent power supply to the electrolyzer or the installation of a buffer tank after the electrolyzer can be employed to maintain a steady feed supply.^94^ Pulsed electrolysis can utilize the buffer tank to provide a consistent feed to the separation equipment.

**Figure 11:**
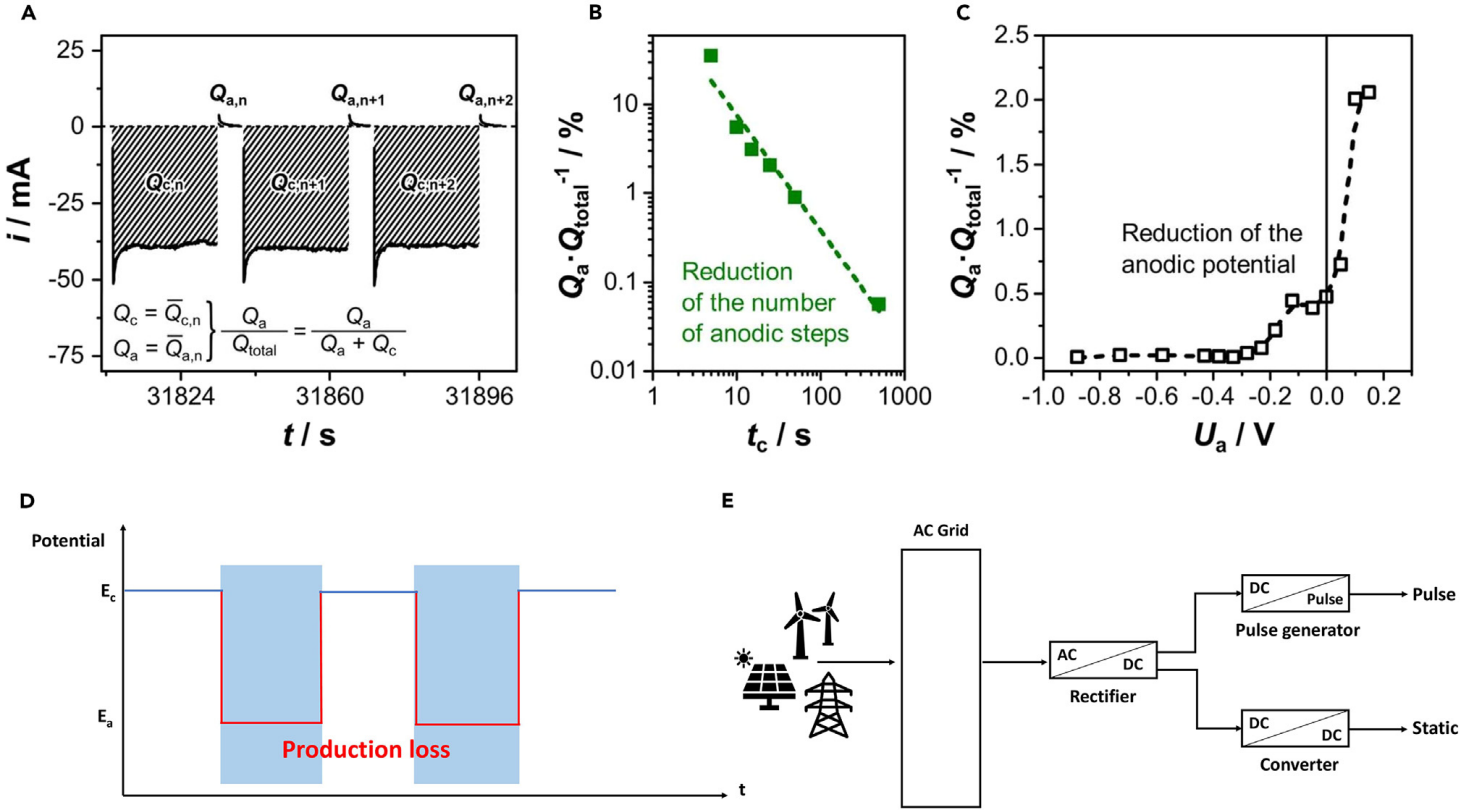
Pulsed CO_2_ electrolysis energy loss (A–C) The calculation of relative anodic charge loss that occurs as a function of cathodic length and anodic potential variation with t_c_ = 25 s, t_a_ = 5 s, U_c_ = −1.6 V, and U_a_ = −0.05 V.^26^ (D) Production loss occurs during the anodic potential (The production does not occur in the areas inside the blue boxes). (E) Power supply systems for static and pulsed electrolysis.

When the CO_2_ electrolysis process operates in an industrial plant, a large-scale power supplement system must be established to supply the necessary power to the electrolyzer. Figure 11E illustrates the general structure of a power supplement system using renewable energy and grid electricity. Energy transmission is typically carried out in AC form to minimize power losses.^95^^,^^96^ The transmitted power is stored in the AC grid and supplied to the CO_2_ electrolysis system. In static electrolysis, the target input voltage is applied through AC/DC rectifiers and DC transducers.^97^^,^^98^ Unlike constant CO_2_ electrolysis, which applies a steady voltage, pulsed electrolysis requires a periodically changing voltage. In the case of pulsed electrolysis, a pulse generator is utilized to convert the DC voltage into the desired pulse profile. Especially since it is not merely about creating a simple pulse profile (e.g., on-off pulse), a system is required that allows the customization of the amplitude and duration of cathodic and anodic potentials in the desired profile.^18^

At industrial scale, pulsed CO_2_ electrolysis demands a supply of relatively low-voltage, high-power energy.^99^ To supply high power to the electrolyzer, a single power converter may not be sufficient to handle the load. In the case of static electrolysis, where multiple power converters are used in parallel to distribute the power, the efficiency of these power converters—which are specific to constant case—typically exceeds 92%–95%.^100^^,^^101^^,^^102^^,^^103^ Consequently, the overall efficiency reduction in the entire conversion process is maintained at about 2%–6%. The efficiency of such power supplement systems can vary depending on their configuration, presenting a critical factor influencing overall cost-effectiveness. Furthermore, these power supplement costs account for approximately 20%–40% of the electrolyzer cost (depending on the power capacity), making them a significant factor.^97^^,^^98^ Thus, it is crucial to compare the difference in the power supplement system between the static and pulsed electrolysis. Despite this significance, the field of research on large-scale pulse power supplement systems remains uncharted. Therefore, research on large-scale pulse power supply systems that meet high efficiency and low-cost requirements is needed.

## Trade-off between production loss and improved performance

In contrast to its economic advantages, pulsed electrolysis has a critical drawback in that it cannot generate product during the anodic potential (Figure 11D). Therefore, charge loss occurs during the period when the potential is applied without the production. To assess the charge loss of pulsed electrolysis, Engelbrecht et al.^26^ investigated how charge loss during the anodic step varies with potential amplitude and length. To quantitatively evaluate this, they defined Q_a_/Q_total_ (where Q_total_ and Q_a_ represent the average total charge and total anodic charge, respectively) as a metric representing the average charge loss relative to the average total charge (Figure 11A). When extending the length of the cathodic pulse (t_c_) from 5 s to 25 s, the anodic charge loss decreased from around 10% to less than 1% (Figure 11B). Additionally, increasing the anodic potential (U_a_) from −1.0 V to 0.15 V (vs. Ag/AgCl) caused charge loss to rise from less than 0.05% to over 2% (Figure 11C). Lee et al.^63^ also examined the charge and electrical energy consumed due to the oxidation state. They applied voltages for the reduction and oxidation steps—which were 0.18 + 1.23 V and 1.22 + 0.00 V, respectively (assuming a zero overpotential for the counter electrode)—with durations of 590 s and 10 s, respectively. Charge consumed during the oxidation step represented only 3.7% of the total charge passed during the reduction step, and electrical energy consumption amounted to a mere 1.9%.

The authors suggested that charge loss due to anodic potential changes is relatively low.^26^^,^^59^^,^^63^ However, a problem arises as production is halted during the pulse-off period (same as anodic duration), leading to a reduction in the amount of product compared to the static process. The decrease in production increases the minimum selling price (MSP) of the product, but it can be offset by the increase in selectivity and stability. Therefore, we plan to identify the cost trade-off between the decrease in production (duty cycle) and the improvement in performance (selectivity, stability) through an economic analysis of the pulsed CO_2_ electrolysis system. Since our model fixes the power input, the production rate varies for each case. Therefore, we calculate the MSP of the product to compare the economic viability with parameter variations. To achieve this, we design a process for single ethylene production and hydrogen as byproduct based on the Na et al.^5^ The detailed process simulation can be found in the Supplemental Information. The process variables for pulsed CO_2_ electrolysis are defined as duty cycle, selectivity, catalyst lifetime, pulse generator ratio, and charge loss ratio. Duty cycle is calculated as the ratio of the cathodic length, representing the proportion of time product is produced during the overall operation time (Equation 8). This value ranges from 0 to 1, with lower values indicating a decrease in annual production compared to static conditions. The cost of the pulse generator is estimated as a ratio of the electrolyzer cost, denoted by the pulse generator ratio. Additionally, a charge loss ratio is set to reflect charge loss due to anodic potential (Equation 9).

**Figure 12:**
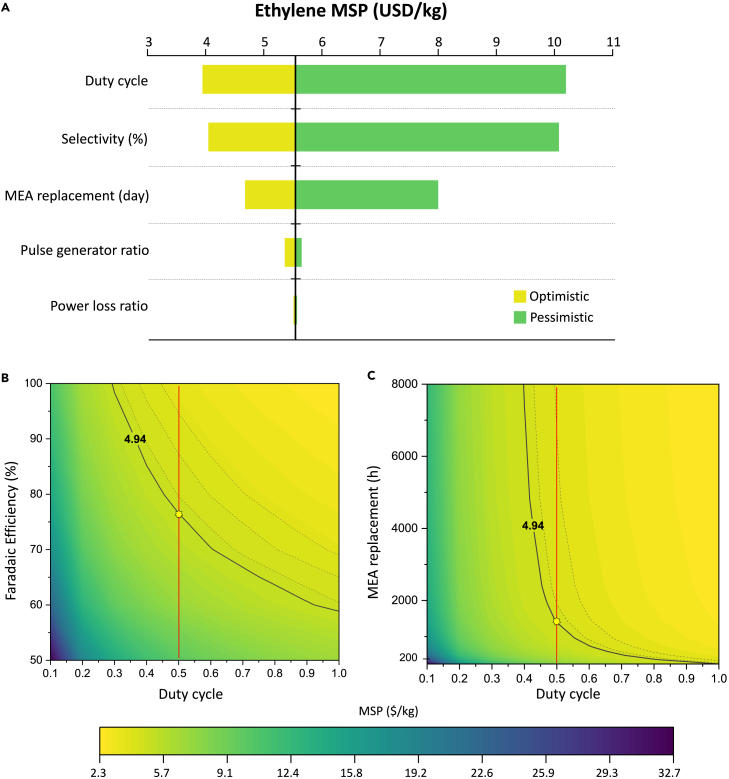
The variation in ethylene’s MSP in pulsed CO_2_ electrolysis (A) Single-variable sensitivity analysis for ethylene production cost. Production cost with (B) selectivity (C) catalyst lifetime as an independent variable. The black solid line represents the cost (MSP: 4.94 $/kg) at the static base (FE: 70%, MEA replacement: 10 days), whereas the red solid line represents the cost variation under the pulse base.

We conduct a single-variable sensitivity analysis to address the following questions: (1) What is the production cost of pulsed CO_2_ electrolysis? (2) What are the main parameters contributing to the changes in cost-effectiveness? (Figure 12A). The ranges for the five pulse parameters to be used in the sensitivity analysis are in Table 2. The base case MSP for ethylene is $5.53/kg; the breakdown of capital and operating costs in the base case is shown in the Figure S2. The pulse generator is estimated to have a similar CAPEX to separation units (PSA, compressor). In terms of OPEX, the electricity consumption decreases relatively due to the duty cycle, whereas the proportion of MEA replacement cost increases due to the short catalyst lifetime. According to sensitivity analysis, we confirm that duty cycle and selectivity are the most significant parameters for production cost. The additional electricity cost attributable to charge loss is relatively small at 1% of OPEX. However, the impact of reduced duty cycle dominates, especially exhibiting an exponential increase between 0.3 and 0.1 (Figure S1). Though, as mentioned earlier, pulsed electrolysis offers the potential for improved selectivity in C_2+_ products and increased catalyst lifetime. To understand this trade-off relationship, we plot MSP variations based on changes in duty cycle, selectivity, and stability from the base conditions (Figures 12B and 12C).

**Table 2:** Range of values for sensitivity analysis

Target product	Ethylene		
Sensitivity parameters	Optimistic	Base	Pessimistic
Selectivity (%)	90	70	50
Duty cycle	0.9	0.5	0.2
Stability (day)	10	30	100
Pulse generator ratio	0.1	0.2	0.3
Charge loss ratio	0.05	0.1	0.15

The red solid line represents the variation in performance under the pulse base condition, indicating that achieving higher performance beyond the crossover point is necessary to be more cost-effective than the static method. As shown in Figure 12B, despite its high selectivity, low duty cycle exhibits higher production cost. This allows us to roughly assess the range of performance that needs to be achieved to offset the decrease in production due to the duty cycle. Catalyst lifetime, as shown in Figure S1, can have a significant impact when it is low, leading to exponential changes in cost. Though, beyond approximately 500 h, the magnitude of change decreases, resulting in less pronounced offset effect compared to selectivity. Consequently, when having a duty cycle between 0.1 and 0.4, the increase in catalyst lifespan cannot offset the reduction in production, making it impossible to reach a lower MSP than static electrolysis (Figure 12C). The effects mentioned earlier will vary depending not only on the electrolyzer performance and the duty cycle but also on the product being produced. Considering the trade-off between performance improvement and production reduction at various process conditions is crucial, and optimizing pulse profiles based on this understanding is essential.

**Figure 13:**
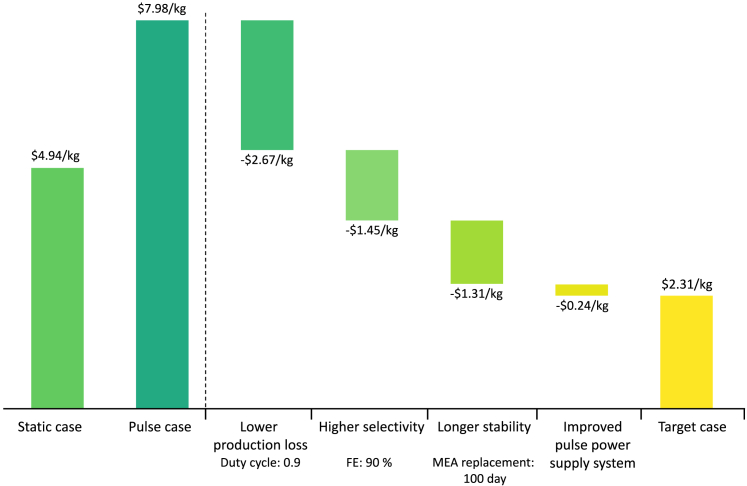
Cost reduction roadmap for pulsed CO_2_ electrolysis to produce ethylene The static and pulse cases have the same performance (FE: 70%, MEA replacement: 10 days), with a duty cycle of 0.5 for the pulse case.

If the static and pulse electrolysis have the same performance, the MSP of ethylene is 1.5 times higher for pulsed electrolysis. Therefore, we illustrate a cost reduction roadmap to achieve higher competitiveness in the form of a waterfall analysis (Figure 13). Sensitivity analysis highlights that achieving high duty cycle and selectivity is crucial for price reduction. Therefore, by increasing the duty cycle to 0.9 and improving selectivity to 90%, we can reduce costs by more than 50% to minimize production losses. Additionally, extending the catalyst lifetime to 100 days reduces MEA replacement costs. We apply the charge loss ratio and pulse generator cost to the optimistic case. As a result, the product cost can be reduced by 70% compared to the baseline and achieve over 45% more cost-effectiveness than the static case. The suggested product cost is slightly higher than market price, but it is anticipated that achieving a higher feasibility is possible through optimization of other economic parameters (e.g., electricity cost, stack cost). However, since the purpose of this paper is to compare the economics between static and pulse CO_2_ electrolysis, other process parameters excluding the five variables are fixed as shown in Table S1.

In this section, we conducted the process design and economic analysis of pulsed electrolysis at the industrial scale. Unlike the static case, pulsed CO_2_ electrolysis requires (1) distinct device to control the discontinuous product stream for downstream process and (2) the establishment of an industrial-scale pulse power supplement system. Additionally, evaluating the impact of duty cycle reduction on annual production is crucial since it affects the economics significantly. Consequently, our analysis of the cost trade-off between production reduction and performance improvement reveals that duty cycle has the greatest impact on the product cost. Therefore, understanding how each cost element changes depending on the chosen pulse profile enables the optimization of pulse profiles beyond the electrolyzer scale, enhancing economic viability at the process scale. However, there are several complement points in this techno-economic analysis. These points are discussed in the limitations of the study.

## Conclusion

In this paper, we review theoretical and computational studies on pulsed CO_2_ electrolysis and present perspectives on the techno-economic aspects of this process. The distinguishing characteristics of the pulse electrolysis include (1) catalyst poisoning inhabitation, (2) surface reconstruction, (3) surface coverage rearrangement, and (4) mass transport limitation, which vary depending on the applied pulse profile. This variability has complex, simultaneously occurring underlying mechanisms. Therefore, through computational methods, we can explore the changes in the mass transport of species at the continuum scale and the reaction mechanisms through DFT calculations. However, there is a limitation in applying DFT calculations to the catalyst’s surface, which changes dynamically according to the pulse characteristics. Finally, we specifically discuss the economic differences between pulsed CO_2_ electrolysis and the conventional constant potential electrolysis and evaluated how these differences affect the process’s relative viability. From a process scale perspective, the key differences lie in the fact that (1) power supplementation and (2) an unsteady-state production control system are necessary and that (3) production loss occurs. While pulse profile modulation can enhance selectivity and stability at the electrolyzer scale, at the process scale, charge loss and production loss occur due to the anodic potential. Therefore, understanding the impact of each factor on the process’s economic viability and selecting pulse profiles with shorter anodic periods and lower anodic voltages while enhancing product selectivity and stability could lead to high economic efficiency in this process.

### Limitations of the study

We are designing a process model and conducting a techno-economic assessment to explore the feasibility of pulsed CO_2_ electrolysis. This model represents a process for producing ethylene from carbon dioxide through pulsed electrolysis. Due to various assumptions made during the techno-economic assessment, there are several limitations.

Firstly, the price of the pulse generator is arbitrarily set based on the IRENA report.^98^ It is necessary to calculate it more specifically, considering factors such as price variations according to power capacity and actual market price. Also, a quantitative analysis of potential power loss that may occur in this pulse power supplement system is omitted. Secondly, the calculation of anodic charge loss should be accurately computed, considering the anodic potential and duration. In this analysis, only the duty cycle ratio of the total charge is considered, suggesting a need for further refinement. Thirdly, an environmental assessment is needed according to the short MEA replacement cycle. We conducted the TEA assuming a relatively short lifespan; it is necessary to understand the influence of these factors on the environment, such as the global warming impact. There are various methods for handling of them, and the environmental effect can vary depending on the situation.^104^^,^^105^ Lastly, the variation in CO_2_ conversion due to the continuous feed injected into the electrolyzer should be considered. In pulsed electrolysis, even during the pulse-off duration, feed is continuously injected, leading to an increase in unreacted CO_2_, which could ultimately lower the purity of the product stream and increase separation costs. To address this issue, considering the CO_2_ conversion variation based on the duty cycle is essential, as this analysis assumes discrete feed injection.

However, since there has been an absence of assessment of pulsed CO_2_ electrolysis at the process scale, we can emphasize the importance of optimizing pulse profiles considering process scale through our study.

## Process simulation and details

Our process model is illustrated in the Figure S3. The model consists of an electrolyzer, gas/liquid separation, CO_2_ capture, and product purification unit. We use the PENG-ROB thermodynamics model and APV 120 and NISTV for materials database. We assume the electrolyzer to be an AEM-based zero-gap MEA. CO_2_ electrolysis is simulated by applying the below equation to the RStoic reactor due to convergence issues with anions in Aspen plus. Therefore, we substitute KOH for hydroxide.

(Equation 5) $$2{CO}_{2}+8H_{2}O+12K\to C_{2}H_{4}+12KOH$$

(Equation 6) $$2H_{2}O+2K\to H_{2}+2KOH$$

(Equation 7) $$4KOH\to O_{2}+2H_{2}O+4K$$

In Aspen plus, it is difficult to simulate changes in production rate and composition based on electrolyzer performance (e.g., current density, CO_2_ conversion, product selectivity), so we address this by using MATLAB. Changes in electrolyzer feed flow rate and production rate due to cell performance are calculated using MATLAB and connected into Aspen plus. The calculation for the CO_2_ electrolysis production rate is outlined in the Supplemental information (section PV-calculation). In this model, since the decrease in CO_2_ conversion due to anodic potential is not considered, we assume that the electrolyzer feed is only supplied when cathodic potential is applied. Water and anion transport through the membrane are simulated by C:AEM1, A:AEM1, and A:AEM2.

(Equation 8) $$\text{Duty}\;\text{cycle}=\frac{\text{cathodic}\;\text{length}}{\text{cathodic}\;\text{length}+\text{anodic}\;\text{length}}$$

(Equation 9) Chargeloss=totalelectrolyzerwork∗chargelossratio∗(1−dutycycle)

In pulsed electrolysis, product is not produced while the anodic potential is applied. Therefore, this must be accounted for in the process model. To address this, the stream coming out of the electrolyzer is split in the simulation to only pass on the amount produced in pulsed electrolysis to the downstream process. Reduction in production rate according to the duty cycle is simulated through B1 and B2 blocks (splitter). Consequently, the downstream process deals only with the decreased production volume. Unreacted CO_2_ is separated by the CO_2_ capture unit. In this model, the process is simply simulated through component splitters.

Product separation (ethylene/hydrogen) is achieved by PSA unit. We used a PSA custom model, which separates ethylene and hydrogen by leveraging the variation in adsorption due to the pressure differential between two vessels, utilizing the adsorption isotherm of Zeolite LiX.^106^ This allows for the calculation of the adsorbent weight based on the input gas flowrate and the purity of the product. We have set the purity of target product to be at least 99%. The weight of adsorbents is a critical factor in determining the CAPEX, including the size of the PSA vessel.

We conduct the techno-economic assessment under Table S1. The electrolyzer cost is estimated based on the DOE H2A analysis for the current central.^98^ The cost of the pulse generator is reflected as a ratio of the electrolyzer cost, referring to a power supply electronics that applies a conventional electrolysis system. Generally, as the power capacity increases, the proportion of the power supply increases, accounting for 20%–40%.^97^^,^^98^ Therefore, assuming the pulse generator price is 20% of the electrolyzer, optimistic and pessimistic scenarios are set at 10% and 30%, respectively. These values are then utilized for techno-economic analysis, with the framework based on Seider et al.^107^ The detailed techno-economic assessment methodology is provided in the Supplemental Information.
