# Supplementary material for: Pulsed electrolysis for CO2 reduction: Techno-economic perspectives
Source: iScience. 2024 Jun 27;27(8):110383. doi: 10.1016/j.isci.2024.110383 (PMC11301079; doi:10.1016/j.isci.2024.110383)
Supplement: Document S1. Figures S1–S3 and Table S1 [file mmc1.pdf]

iScience, Volume *27*

Supplemental information

Pulsed electrolysis for CO_2 reduction: Techno-economic perspectives

You Lim Chung, Sojin Kim, Youngwon Lee, Devina Thasia Wijaya, Chan Woo Lee, Kyoungsuk Jin, and Jonggeol Na

Pulsed CO_2 electrolysis techno-economic analysis

1) Process modeling

The purpose of this process is to observe the change in ethylene's MSP according to variables (e.g., duty cycle, selectivity, catalyst lifetime) when the power is fixed. Other parameters are fixed based on other CO_2 electrolysis techno economic analysis study.[1,2] (Table S1) The production rate is calculated under the given conditions, and simulations are conducted using Aspen Plus simulators. Based on the obtained heat and mass balances, an economic evaluation is performed. This modeling and analysis process is conducted based on Na et al.[3]

$$\text{Charge loss}=\text{total electrolyzer work}*\text{charge loss ratio}*(1\text{-duty cycle}) \qquad \text{(Equation S1)}$$

$$\text{Duty cycle}=\frac{\text{cathodic length}}{\text{cathodic length}+\text{anodic length}} \qquad \text{(Equation S2)}$$

In pulsed electrolysis, product is not produced while the anodic potential is applied. Therefore, this must be accounted for in the process model. To address this, the stream coming out of the electrolyzer is split in the simulation to only pass on the amount produced in pulsed electrolysis to the downstream process. The electricity and feed of the electrolyzer are reflected in the operating cost only when the cathodic potential is applied. Additionally, charge loss due to anodic potential is accounted for using (Equation S1). The cost of the pulse generator is reflected as a ratio of the electrolyzer cost, referring to a power supply system that applies a constant voltage. Generally, as the power capacity increases, the proportion of the power supply increases, accounting for 20-40%.[4,5] Therefore, assuming the pulse generator price is 20% of the electrolyzer, optimistic and pessimistic scenarios are set at 10% and 30%, respectively. In the downstream process, for the cathode, as ethylene is a gas product, it consists of gas/liquid separation (flash drum) for electrolyte separation, CO_2 capture (CCS), and pressure swing adsorption (PSA) for H_2 separation. For the anode, there is gas/liquid separation for O_2

separation.

Parameter	Value
Current density	1000 mA/cm^2
Cell voltage	2.9 V
Conversion	0.3
Electrolyzer cost	$550/kW
PV power (fixed)	100 MW
Electricity cost	$0.03/kWh
CO_2 purchase price	$0.04/kg
Operating time	8000 h

Table S1. Process assumptions for Pulsed CO_2 electrolyzer, Related to Figure 12.

2) Techno-economic analysis

We provide an example of producing ethylene through pulsed CO_2 electrolysis. Chemical stoichiometric numerical simulations are conducted using the production rates calculated via PV-EC calculations. Based on the DOE H2A analysis for the current central, the stack cost is set at $550/kW.[4] The reference electrolyzer operates at 1.75 V and 0.4 A/cm^2, with an installation factor of 1.12. Using these conditions, the electrolyzer cost is estimated by Na et al.[3] methodology. These values are then utilized for techno-economic analysis, with the framework based on Seider et al.[6] The examples of capital and operating costs are results from base condition pulsed CO_2 electrolysis. Additionally, the balance of plant is included in the electrolyzer cost.

<PV-EC calculation>

Actual amount of produced electricity from photovoltaic system (PV power=100 MW):

$$\overline{E}_{PV}=E_{farm}\eta_{CF}=100\ \text{MW}\times 0.24\times 10^{6}\,\frac{\text{W}}{\text{MW}}=24.00\times 10^{6}\ \text{W}.$$

Total required electrolyzer cell area A_{cell}:

$$A_{cell}=\frac{\overline{E}_{PV}}{(E^{0}_{anode}+\eta_{anode}-E^{0}_{cathode}+\eta_{cathode})\times CD}=\frac{24.00\times 10^{6}\ \text{W}}{(0.401\text{-}(\text{-}0.743)+1.8)\ \text{V}\times 1000\frac{\text{mA}}{\text{cm}^{2}}}\times 10^{3}\,\frac{\text{mA}}{\text{A}}\times 10^{-4}\,\frac{\text{m}^{2}}{\text{cm}^{2}}=814\ \text{m}^{2}.$$

The production rate of C_2H_4:

$$\dot{n}^{out}_{cathode}=\frac{CD\times FE_{cathode}\times A_{cell}}{F\times z_{cathode}}\times\text{duty cycle}=\frac{1000\frac{\text{mA}}{\text{cm}^{2}}\times 0.7\times 814\ \text{m}^{2}}{96485\frac{\text{C}}{\text{mol}}\times 12}\times 10^{-3}\,\frac{\text{A}}{\text{mA}}\times 10^{4}\,\frac{\text{cm}^{2}}{\text{m}^{2}}\times 0.5=2.45\,\frac{\text{mol}}{\text{sec}}.$$

The production rate of H_2:

$$\dot{n}^{out}_{cathode}=\frac{CD\times FE_{cathode}\times A_{cell}}{F\times z_{cathode}}\times\text{duty cycle}=\frac{1000\frac{\text{mA}}{\text{cm}^{2}}\times 0.3\times 814\ \text{m}^{2}}{96485\frac{\text{C}}{\text{mol}}\times 2}\times 10^{-3}\,\frac{\text{A}}{\text{mA}}\times 10^{4}\,\frac{\text{cm}^{2}}{\text{m}^{2}}\times 0.5=6.35\,\frac{\text{mol}}{\text{sec}}.$$

The production rate of anode:

$$\dot{n}^{out}_{anode}=\frac{CD\times FE_{anode}\times A_{cell}}{F\times z_{anode}}\times\text{duty cycle}=\frac{1000\frac{\text{mA}}{\text{cm}^{2}}\times 1\times 814\ \text{m}^{2}}{96485\frac{\text{C}}{\text{mol}}\times 4}\times 10^{-3}\,\frac{\text{A}}{\text{mA}}\times 10^{4}\,\frac{\text{cm}^{2}}{\text{m}^{2}}\times 0.5=10.55\,\frac{\text{mol}}{\text{sec}}.$$

<Capital cost>

Total bare-module costs for equipments		
Electrolyzer		$3,417,300
Flash		$8,764
Compressor		$629,300
PSA		$335,990
HX		$4,539
Pulse generator		$683,460
**Total bare-module investment, TBM**		**$5,079,400**
Cost of site preparation	0.2×TBM	$1,015,880
**Total of direct permanent investment, DPI**		**$21,470,451**
Cost of contingencies and contractor's fee	0.15×DPI	$914,280
**Total depreciable capital, TDC**		**$7,009,500**
Cost of land	0.02×TDC	$140,790
Cost of Plant startup	0.1×TDC	$700,950
**Total permanent investment, TPI**		**$7,850,600**

<Operating cost>

**Cost Factor**	**Annualized Cost ($)**
**Feedstocks**	
Electrolyte	$257,100
CO_2	$541,410
Water	$53
**Utilities**	
Electricity	$2,040,200
Refrigeration	$309
CO_2 capture cost	$874,580
**Operations (labor-related) (O)**	
Direct wages and benefits (DW&B)	$320,000
Direct salaries and benefits	15% of DW&B

Operating supplies and services	6% of DW&B
Technical assistance to manufacturing	$180,000
Control laboratory	$195,000
**Maintenance (M)**	
Wages and benefits (MW&B)	$245,330
Salaries and benefits	25% of MW&B
Materials and services	100% of MW&B
MEA replacement (catalyst lifetime=30 day)	$1,927,000
Stack replacement (for 7 year)	$87,873
Maintenance overhead	5% of MW&B
**Operating overhead**	
General plant overhead	7.1% of M&O-SW&B
Mechanical department services	2.4% of M&O-SW&B
Emplyee relations department	5.9% of M&O-SW&B
Business services	7.4% of M&O-SW&B
**Property taxes and insurance**	2% of TDC
**Depreciation**	
Direct plant	8% of (TDC-1.18alloc)
Allocated plant	6% of 1.18alloc
**COST OF MANUFACTURES (COM)**	**$7,910,100**
**General Expenses**	
Selling (or transfer) expense	3% (1%) of sales
Direct research	4.8% of sales
Allocated research	0.5% of sales
Administrative expense	2.0% of sales
Management incentive compensation	1.25% of sales
**TOTAL GENERAL EXPENSES (GE)**	**$1,133,200**
**TOTAL PRODUCTION COST (C)**	**$9,043,300**

Cash flow analysis is performed with a plant lifespan of 25 years, a construction period of 2 years, 7-year MACRS depreciation, a 10% nominal interest rate, and a 40% income tax rate to calculate the net present value (NPV). The MSP of the product is calculated to achieve an NPV of 0. As a result, the MSP of ethylene produced by pulsed CO_2 electrolysis under the base conditions is calculated to be $5.53/kg, indicating a considerably high value. In terms of capital cost, the electrolyzer accounts for approximately 70%, followed by the pulse generator at 15%. Additionally, it can be observed that the proportion of electricity cost relative to static is reduced due to the duty cycle. MEA replacement cost is calculated to be high due to the low catalyst lifespan of 30 days.

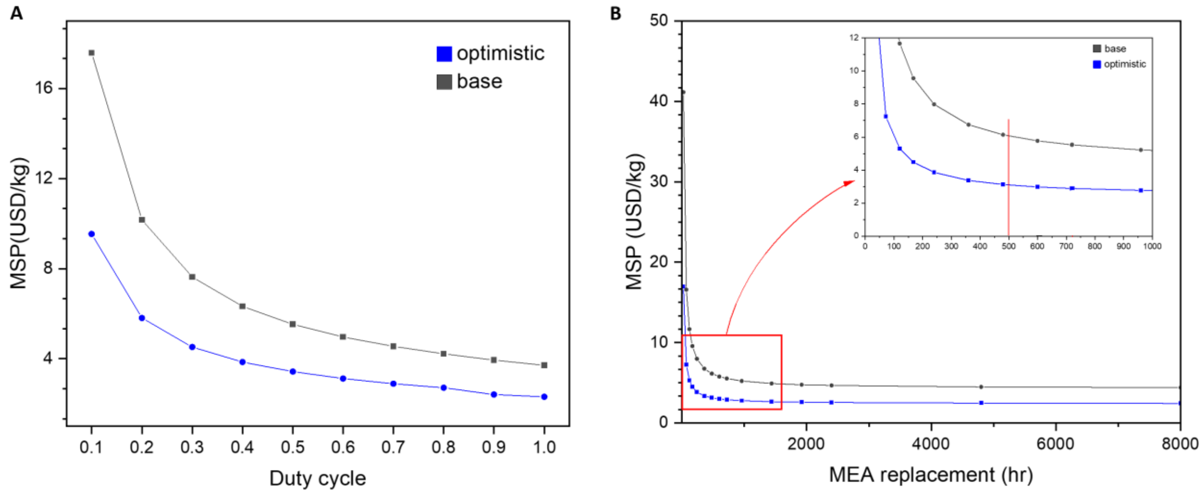

Figure S1. Change in ethylene MSP, Related to Figure 12. MSP change according to (A) duty cycle and (B) MEA replacement**.** Blue line represents the pulse optimistic case, the gray line represents the pulse base case.

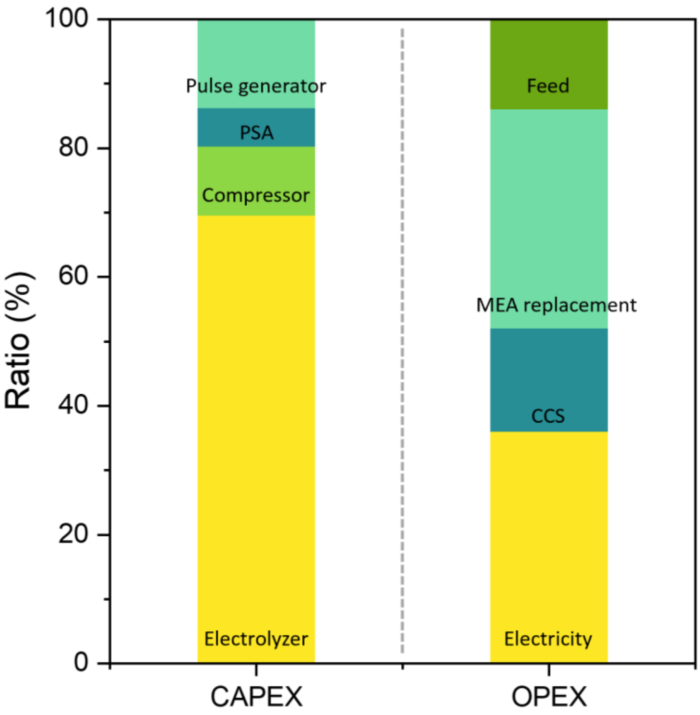

Figure S2. The cost breakdown of each unit to CAPEX and OPEX in terms of ratio, based on pulsed CO_2 electrolysis base conditions, Related to Figure 12.

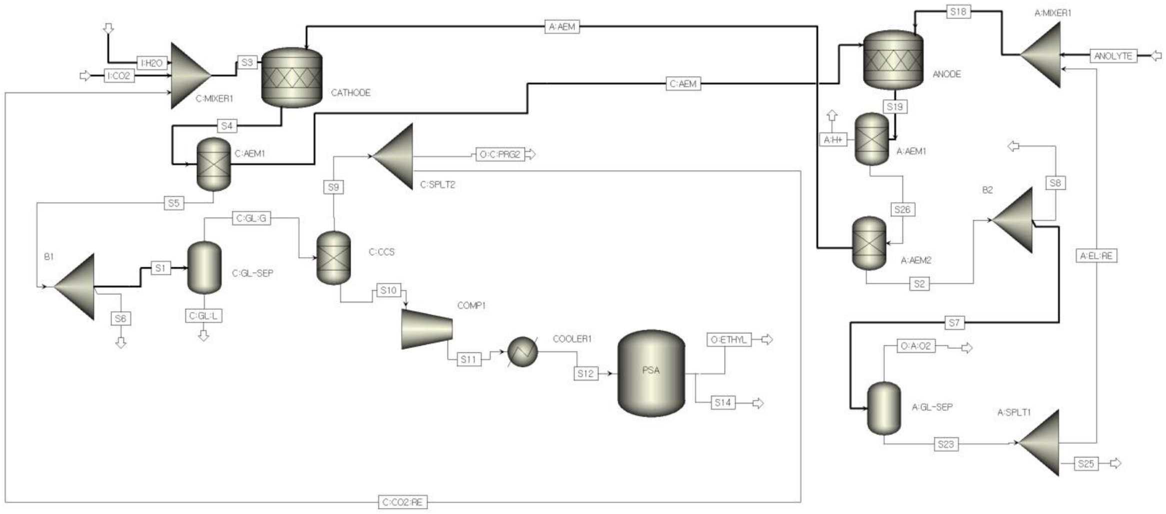

Figure S3. Pulsed CO_2 electrolysis process flow diagram in Aspen plus, Related to Figure 10. The bold solid line represents the stream that directly varies according to cell performance (FE, duty cycle, conversion, etc).

<Stream table at pulse base condition>

Name	Unit	A:AEM	A:EL:RE	A:H+	ANOLYTE	C:AEM	C:CO2:RE	C:GL:G	C:GL:L
T	K	298.15	298.15	298.15	298.15	298.15	298.15	298.15	298.15
P	atm	1	1	1	1	1	1	1	1
F	kmol/sec	0.065	0.025	12.000	1.060	11.000	0.012	0.021	0.0002
z[H2]	mol frac	0	0	0	0	0	0	0.304	0
z[H2O]	mol frac	1	1	0	0.057	0	0.045	0.026	1
z[CO2]	mol frac	0	0	0	0	0	0.955	0.552	0
z[O2]	mol frac	0	0	0	0	0	0	0	0
z[C2H4]	mol frac	0	0	0	0	0	0	0.118	0
z[KOH]	mol frac	0	0	0.083	0	0.099	0	0	0
z[K+]	mol frac	0	0	0.917	0.943	0.901	0	0.	0

Name	Unit	I:CO2	I:H2O	O:A:O2	O:C:PRG2	O:ETHYL	S1	S2	S3
T	K	298.15	298.15	298.15	298.15	298.15	298.15	298.15	298.15
P	atm	1	1	1	1	1.28	1	1	1
F	kmol/sec	0.021	11.001	0.011	0	0.002	0.021	0.083	11.034
z[H2]	mol frac	0	0	0	0	0.004	0.301	0.746	0
z[H2O]	mol frac	0	0	0.026	0	0	0.037	0	0
z[CO2]	mol frac	1	0	0	0	0	0.545	0.254	0.003
z[O2]	mol frac	0	0	0.974	0	0	0	0	0
z[C2H4]	mol frac	0	0	0	0	0.996	0.117	0	0
z[KOH]	mol frac	0	0.091	0	0	0	0	0	0.091
z[K+]	mol frac	0	0.909	0	0	0	0	0	0.906

Name	Unit	S4	S5	S6	S7	S8	S9	S10	S11
T	K	298.15	298.15	298.15	298.15	298.15	298.15	298.15	740.71
P	atm	1	1	1	1	1	1	1	27.3
F	kmol/sec	11.042	0.042	0.021	0.042	0.042	0.012	0.009	0.009
z[H2]	mol frac	0.001	0.301	0.301	0	0	0	0.720	0.720
z[H2O]	mol frac	0	0.037	0.037	0.746	0.746	0.045	0	0
z[CO2]	mol frac	0.002	0.545	0.545	0	0	0.955	0	0
z[O2]	mol frac	0	0	0	0.254	0	0	0	0
z[C2H4]	mol frac	0	0.117	0.117	0	0.254	0	0.280	0.280
z[KOH]	mol frac	0.098	0	0	0	0	0	0	0
z[K+]	mol frac	0.898	0	0	0	0	0	0	0

Name	Unit	S12	S14	S18	S19	S23	S25	S26
T	K	298.15	298.15	298.15	298.15	298.15	298.15	298.15
P	atm	1	1.28	1	1	1	1	1
F	kmol/sec	0.009	0.007	0.084	12.148	0.031	0.006	0.148

z[H2]	mol frac	0.720	0.959	0	0	0	0	0
z[H2O]	mol frac	0	0	1	0.010	1	1	0.857
z[CO2]	mol frac	0	0	0	0	0	0	0
z[O2]	mol frac	0	0	0	0.002		0	0.143
z[C2H4]	mol frac	0.280	0.041	0	0	0	0	0
z[KOH]	mol frac	0	0	0	0.082	0	0	0
z[K+]	mol frac	0	0	0	0.906	0	0	0

References

1. Jouny, M., Luc, W., and Jiao, F. (2018). General techno-economic analysis of CO2 electrolysis systems. Industrial & Engineering Chemistry Research *57*, 2165-2177.
2. Shin, H., Hansen, K.U., and Jiao, F. (2021). Techno-economic assessment of low-temperature carbon dioxide electrolysis. Nature Sustainability *4*, 911-919.
3. Na, J., Seo, B., Kim, J., Lee, C.W., Lee, H., Hwang, Y.J., Min, B.K., Lee, D.K., Oh, H.-S., and Lee, U. (2019). General technoeconomic analysis for electrochemical coproduction coupling carbon dioxide reduction with organic oxidation. Nature communications *10*, 5193.
4. Taibi, E., Miranda, R., Carmo, M., and Blanco, H. (2020). Green hydrogen cost reduction.
5. Mayyas, A.T., Ruth, M.F., Pivovar, B.S., Bender, G., and Wipke, K.B. (2019). Manufacturing cost analysis for proton exchange membrane water electrolyzers. National Renewable Energy Lab.(NREL), Golden, CO (United States).
6. Seider, W.D., Lewin, D.R., Seader, J., Widagdo, S., Gani, R., and Ng, K.M. (2017). Product and process design principles: synthesis, analysis, and evaluation (John Wiley & Sons).
